# Information needs in people with diabetes mellitus: a systematic review

**DOI:** 10.1186/s13643-018-0690-0

**Published:** 2018-02-14

**Authors:** Lisa Biernatzki, Silke Kuske, Jutta Genz, Michaela Ritschel, Astrid Stephan, Christina Bächle, Sigrid Droste, Sandra Grobosch, Nicole Ernstmann, Nadja Chernyak, Andrea Icks

**Affiliations:** 1 Institute for Health Services Research and Health Economics, Centre for Health and Society, Faculty of Medicine, Heinrich Heine University, Moorenstraße 5, 40225 Düsseldorf, Germany; 2 Institute for Health Services Research and Health Economics, German Diabetes Center (DDZ), Leibniz Institute for Diabetes Research, at Heinrich Heine University Düsseldorf, Auf’m Hennekamp 65, 40225 Düsseldorf, Germany; 3 Center for Health Communication and Health Services Research (CHSR), Department for Psychosomatic Medicine and Psychotherapy, University Hospital Bonn, Sigmund-Freud-Str. 25, 53127 Bonn, Germany; 4 German Center for Diabetes Research (DZD), Ingolstädter Landstraße 1, 85764 Neuherberg, Germany

**Keywords:** Diabetes, Information needs, Information-seeking behaviour, Systematic review

## Abstract

**Background:**

The purpose of this study was to identify and analyse currently available knowledge on information needs of people with diabetes mellitus, also considering possible differences between subgroups and associated factors.

**Methods:**

Twelve databases including MEDLINE, EMBASE and the Cochrane Library were searched up until June 2015. Publications that addressed self-reported information needs of people with diabetes mellitus were included. Each study was assessed by using critical appraisal tools, e.g. from the UK National Institute for Health and Care Excellence. Extraction and content analysis were performed systematically.

**Results:**

In total, 1993 publications were identified and 26 were finally included. Nine main categories of information needs were identified, including ‘treatment-process’, ‘course of disease’, ‘abnormalities of glucose metabolism’ and ‘diabetes through the life cycle’. Differences between patient subgroups, such as type of diabetes or age, were sparsely analysed. Some studies analysed associations between information needs and factors such as participation preferences or information seeking. They found, for example, that information needs on social support or life tasks were associated with information seeking in Internet forums.

**Conclusion:**

Information needs in people with diabetes mellitus, appear to be high, yet poorly investigated. Research is needed regarding differences between diverse diabetes populations, including gender aspects or changes in information needs during the disease course.

**Systematic review registration:**

The review protocol has been registered at Prospero (CRD42015029610).

**Electronic supplementary material:**

The online version of this article (10.1186/s13643-018-0690-0) contains supplementary material, which is available to authorized users.

## Background

Diabetes mellitus (DM) is a highly prevalent chronic disorder. People with DM have to perform comprehensive self-management interventions to achieve good diabetes control [1]. In order to make adequate decisions concerning their illness, a sufficient level of disease-related information is required [2]. In fact, people with DM communicate a particularly high need for information, higher than people with cancer or cardiovascular diseases, for example [3, 4]. However, it seems that people with DM do not feel adequately informed about their condition or regarding medication use [3]. Although the importance of an appropriate needs-driven information supply is unquestioned, and a large amount of diabetes information exists, there seems to be limited knowledge about information needs (IN) of people with DM considering different patient subgroups, as well as IN of people with DM in phases of the disease that may affect the need for certain information [5–7]. To provide needs-driven information, deeper insight into the perspectives of people with DM is urgently needed. This is particularly true since it has been suggested that information supply, self-management and health outcomes could be improved if more were known about the perspectives and needs of those concerned [8, 9].

This systematic review aims to identify and analyse currently available knowledge on the IN of people with DM, also considering possible differences between subgroups and associated factors.

## Methods

This systematic review was performed in line with the quality requirements of the PRISMA guideline (available as Additional file 1) [10]. The review protocol has been registered at PROSPERO (CRD42015029610).

As stated in the review protocol, we searched in MEDLINE, EMBASE, CINAHL, ScienceDirect, the Cochrane Library, Web of Science, PsycINFO, CCMed, ERIC and Journals@OVID, Deutsches Ärzteblatt and Karlsruher virtueller Katalog. Publications were included that had been published from the inception of each database up to June 2015 (see Appendix 1 and Appendix 2) [11] with a German or English title and abstract and a full text in any language.

### Search strategy

The search strategy was set up using database-specific vocabularies (MeSH, EMTREE) and additional free-text terms (see Appendix 1 and Appendix 2) [11]. The search algorithm was crosschecked by experienced reviewers (S.K., A.S.) and piloted by comparing results of the search strategy in MEDLINE with core references that were identified by pre-search activities. Search terms for IN included ʻinformation needʼ, ʻknowledge needʼ, ʼwish or desire of informationʼ, ʻinformation preferenceʼ and ‘request for information’. Search terms for DM included: ʻdiabetesʼ, ʻdiabeticʼ, ʻniddmʼ, ʻiddmʼ, ʻt2dmʼ, ʻt1dmʼ, ʻprediabetesʼ, ʻprediabeticʼ, ʻpre-diabetesʼ, ʻpre-diabeticʼ and ʻimpaired glucoseʼ. For further details see Appendix 1 and Appendix 2.

### Inclusion and exclusion criteria

Studies that analysed self-reported IN of people with DM (any type) as a primary or secondary research aim were included. IN are defined as: ʻRecognition that their (people’s) knowledge is inadequate to satisfy a goal, within the context/situation that they find themselves at a specific point in the time [12].’ Original qualitative, quantitative or mixed-methods studies were included. Systematic and narrative reviews, meta-analyses and qualitative meta-syntheses were also included.

Studies reporting the IN of relatives or healthcare professionals were excluded, as well as studies where relatives or healthcare professionals reported IN of people with DM. Publications without available references, letters/short reports, abstracts, editorials, comments or discussion papers were excluded.

### Study selection process

Inclusion and exclusion criteria were pre-tested on 380 records and finally discussed (L.B., J.G., S.K.). Then, two reviewers (L.B., J.G.) independently selected the articles, first by title and abstract and thereafter by full text. All decisions were checked by two other reviewers (S.K., M.R.). Unclear decisions were resolved by an additional reviewer (A.I.).

Full texts were screened with the aim of identifying further original studies using backward citation tracking.

### Data extraction and synthesis

A data extraction sheet was developed following the requirements of Cochrane [10].We extracted the type of information needed by people with DM and, if investigated, by different subgroups, such as type of DM and age. A content analysis was conducted, developing categories according to the topics of the review questions, in particular, to assess the reported and analysed types of information needed. Furthermore, IN-associated factors were extracted and analysed via a content analysis. Codings were developed inductively (L.B.) [13] using a coding protocol and revised critically (S.K., A.I.).

All relevant publications were described according to the following predefined categories: author, date, methods, findings, associated factors and result of the critical appraisal of the study quality. Furthermore, the main categories of IN were described, as well as those related to subgroups of people with DM and the associated factors. We describe the studies stratified for those analysing IN as a primary and a secondary outcome, as well as those with a qualitative, quantitative, or mixed-method study design.

### Critical appraisal

Each study was critically appraised separately (L.B., S.K., J.G., M.R.) by using design-specific critical appraisal tools from the UK National Institute for Health and Care Excellence (NICE) [14]. The study’s quality was described as follows: ‘(++) All or most of the checklist criteria have been fulfilled, where they have not been fulfilled the conclusions are very unlikely to alter. (+) Some of the checklist criteria have been fulfilled, where they have not been fulfilled, or not adequately described, the conclusions are unlikely to alter. (-) Few or no checklist criteria have been fulfilled and the conclusions are likely or very likely to alter.’ [14]. Mixed methods were analysed by the Mixed Methods Appraisal Tool (MMAT)–Version 2011 [15]. The critical appraisal for mixed-methods studies includes whether the mixed-methods design was appropriate and whether the integration was relevant to address the research question (objective). The criteria also consider whether limitations are considered, associated with this integration, e.g. whether the divergence of qualitative and quantitative data (or results) in a triangulation design was appropriate [15].

## Results

**Fig. 1:**
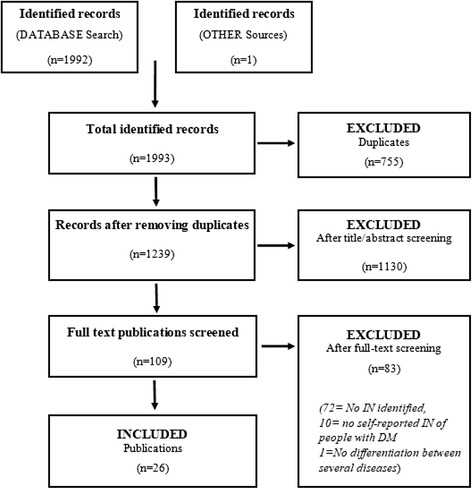
Study Selection Process

In total, 1993 publications were identified that had been published up to June 2015 (Fig. 1), of which 26 publications (*n* = 25 studies) reporting diabetes-related IN of people with DM were finally included (Table 1). The sample sizes varied from 11 to 1609 participants with DM.

**Table 1:** Overview—included studies

Author/year	Methods	Sample size^a^	Population characteristics			Outcomes	Aims/findings	Associated factors	Critical appraisal	Number of criteria
			DM type	Sex^a^	Age^a^					
*In (primary outcome)*										
Qualitative studies										
Lamberts et al. 2010 [16]*	Focus group	*n* = 11	Type 2 DM (T2DM)	f (*n* = 13), m (*n* = 19)	18–80	Information needed and provided according to patients starting oral T2DM medication	The study explored the information needs of patients who have recently started treatment with oral antidiabetics and analysed the provision of information. The study showed that patients are in need of diabetes-medication information such as drug-related issues.	x	+	11/12
Lee et al. 2007 [17]*	Interview	*n* = 24	T2DM	f (*n* = 6), m (*n* = 18)	44–79	Knowledge of diabetes and prescribed medicines; experiences with medicines information; consumer-specific written medicines information needs; written leaflets on medicine information needs	A ‘Consumer Involvement Cycle’ is developed to assist researchers, and to analyse perspectives and needs on medicines information to develop written medicines information. The study identified a lack of written medicine information for people with diabetes, who wish for specific information about their medicine (mechanisms of action, administering instructions, drug-related issues)		+	11/12
Olsen Roper et al. 2009 [19]	Website evaluation	*n* = 340 (messages)	Type 1 DM (T1DM)	f (79%), m (21%) (sex was identified in 48,5% of all messages)	11–19	Reasons for posts; topics of requests; reliability and coding issues; forum differences; gender differences; age and duration of illness	This study explored messages posted on public web-based forums. The people with diabetes expressed information needs regarding the consequences of the disease, social support and life tasks.	x	+	10/12
Ravert et al. 2004 [18]	Interview	*n* = 58	T1DM	f (*n* = 37), m (*n* = 21)	8–18	Existing knowledge about diabetes and information needs about diabetes	This study explored information needs of children with DMT1 among other things concerning diabetes care, its pathophysiology, consequences, treatment and possible cure.	x	+	11/12
Savage et al. 2009 [20]	Focus group	*n* = 13	T1DM	f (*n* = 9), m (*n* = 4)	26–44	Preferred content and delivery mode of education and information	This study showed that the main information need of people with DMT2 between the ages of 25 to 45 was related to adequate information on managing diabetes themselves, e.g. medication, preventing diabetes, pregnancy and emergency management not sufficiently covered at present.		+	11/12
Van Esch et al. 2010 [22]	Website evaluation	*n* = 158	T1DM, T2DM, Gestational DM (GDM), Maturity onset diabetes of the young (MODY)/ Not defined	f, m (no number available for people with DM)	< 20–> 41	Information needs of online consumers (genetics and diabetes)	This study identified information needs about the role of inheritance in diabetes.	x	_	7/12
Wilson et al. 2013 [25]	Questionnaire	*n* = 30	T1DM, T2DM	f (*n* = 16), m (*n* = 14)	22–64	Access; information type;	The study explored the method preferred by people with diabetes to access information about their condition, and what type of information they require.		_	1/12
Quantitative studies										
Duggan et al. 2008 [4]*	Interview	*n* = 117	Not defined (and other diseases)	f, m (no number available for peole with DM)	55.5–60.3	Relationships between information needs, diagnosis and disease	The study showed that different diagnoses and diseases are associated with different medicine information needs.		**+**	2PP, 4P, 4M, 0NR, 9NA
Whitford et al. 2013 [24]	Focus groups and semistructured interviews (within a randomised controlled trial)	*n* = 29 (support groups)	T2DM	NR	NR	Information needs of participants with T2DM	This explored the use of a system of patient-generated ‘frequently asked questions’ in order to gain insight into the information needs of participants.		_	1PP, 3P, 4M, 1NR, 10NA
Whetstone et al. 2014 [23]	Interview	*n* = 21	T2DM	f (*n* = 15), m (*n* = 6)	38–79	Kept health information and information needs	This study explored information behaviour and information needs.	x	+	3PP, 3P, 4M, 1NR, 8NA
Mixed-method studies										
Beeney et al. 1996 [3]	Interview + questionnaire	*n* = 1145	T1DM, T2DM	f (*n* = 573), m (*n* = 572)	39.9 ± 19;64.2 ± 12	Information needs and emotional support	They study explored patient information needs for emotional support and information preferences.	x	**_**	5/21 (11 NA)
Sparud-Lundin et al. 2011 [21]*	Questionnaire	*n* = 105	T1DM	f (*n* = 105)	≤ 30–36 ≥	Socio demographic factors; use of the internet (information seeking and communication); diabetes-related issues and specific questions on needs in relation to childbearing; Expectations of web-based support	This study explored the internet use, the needs and expectations of web-based information and communication. Information needs were expressed regarding diabetes-related aspects, regarding e.g. pregnancy, childbirth, and parenthood.	x	_	5/21 (9 NA)
St. Jean 2012 [8] 2014 [9]	Interview, questionnaire, card sorting, timeline	*n* = 34	T2DM	f (*n* = 20), m (*n* = 14)	≥ 18	Changes across the time: information seeking and use; awareness and capability of articulating information needs; usefulness of sources and types of diabetes-related information	This study explored information behaviour and its changes across time, and identified different content types of diabetes-related information needs, e.g. risk factors, medication, exercise.	x	+	7/21 (13 NA)
*In (secondary outcome)*										
Qualitative studies										
Goldman et al. 2008 [26]*	Interview	*n* = 36	Not defined	f (*n* = 22), m (*n* = 14)	20–61+	Patients’ opinions about automated speech-recognition telephone technology	While developing an automated telephone outreach intervention for people with diabetes, the study obtained IN of patients on nutrition and dietary advice, consequences and blood glucose control.		+	10/12
Hjelm et al. 2008 [27]*	Interview	*n* = 23	GDM	f (*n* = 23)	23–41	Beliefs about health and health care	While exploring beliefs about health, illness and healthcare in women with Gestational Diabetes Mellitus (GDM), further study results identified IN about GDM and its treatment.		++	12/12
Lindenmeyer et al. 2013 [28]*	Interview	*n* = 20	T2DM	f (*n* = 8), *m* (*n* = 12)	40–82	[28] awareness; interaction with dental health professionals and information exchange; information preferences	The study explored the awareness of people with type 2 diabetes, how they communicate with dentists and professionals (primary care), and preferences of how to receive care and information related to oral health.		+	11/12
McCorry et al. 2012 [29]*	Interview	*n* = 14	T1DM	f (*n* = 14)	21–38	Attitude toward pregnancy planning and pre-conception	The study explored attitudes toward pregnancy planning and antenatal care. IN of women with T1DM concerning antenatal care, pregnancy and diabetes management in this time.		++	12/12
Meyfroidt et al. 2013 [30]*	Focus group	*n* = 21	T2DM	f (*n* = 7), m (*n* = 14)	41–85	Use of information sources; information seeking; problems encountered by the patients	While obtaining data to determine how people with diabetes seek and use information sources for their diet, further results identified IN concerning food characteristics.		++	12/12
Peel et al. 2004 [31]	Interview	*n* = 40	T2DM	f (*n* = 19), m (*n* = 21)	21–7	‘Suspected diabetes’ route to diagnosis; ‘illness’ route to diagnosis; ‘routine’ route to diagnosis; information provision at diagnosis; overall emotional reactions to diagnosis	During research on patients’ views on information provision at the time of diagnosis, the study identified the need for information on course of disease and its consequences, diabetes management and advice on nutrition.		+	9/12
Wilkinson et al. 2014 [36] *	Interview	*n* = 47	T2DM	f (*n* = 22), m (*n* = 25)	34–85	Diagnosis of diabetes; symptoms; access; experience of diabetes services; current health; self-management/support	The study explored the quality of diabetes care and identified IN on, for example, diet, risk and complications explained.		_	6/12
Quantitative studies										
Chen et al. 2012 [32]	Web based blog analysis	*n* = 516	T1DM	NR	NR	Patient experience (emotional, temporal)	The study explored online discussion forums for three conditions: breast cancer, T1DM and fibromyalgia. It showed that many people with T1DM addressed topics of diabetes management. However, they were also interested in website references, sharing experiences and support.		**_**	0PP, 1P, 8 M, 0NR, 10NA
Hajos et al. 2011 [34]	Questionnaire	*n* = 1609	T2DM	f (*n* = 660), m (*n* = 949)	51.4 ± 12.5	Seriousness of their diabetes, diabetes-related distress, worries about complications, need for care improvement	The study explored the extent to which physicians understand T2DM, e.g. patients’ perceptions of seriousness and emotional distress, and needs for care improvement. The study showed that people need more information about treatment options, where to get support and the newest information.		+	1PP, 7P, 1 M, 0NR, 9NA
Robertson et al. 2005 [35]	Questionnaire	*n* = 70	T1DM, T2DM	f (*n* = 27), m (*n* = 43)	16–79	Sources and adequacy of information	This study explored the sources of information and their adequacy for supplying diabetes information. The people with diabetes expressed a lack of information about their condition.		+	1PP, 5P. 3 M, 1NR, 9NA
Mixed-method studies										
Frandsen et al. 2002 [33]	Interview + questionnaire	*n* = 123	T2DM	f (*n* = 59), m (*n* = 64)	45–60	Issues and barriers relating to patient compliance	The study explored issues and barriers relating to patient compliance and showed that the people with T2DM want more information about their condition.		_	0/21 (19 NA)
Mühlhauser et al. 1988 [6]*	Interview + questionnaire	*n* = 37	T1DM	f (*n* = 13), m (*n* = 24)	38 ± 9	Blood pressure control (compliance)	The study explored the degree of blood pressure control and identified the need for more information about high blood pressure.		+	4/21 (9 NA)

*Quantitative or qualitative studies, mixed-method studies (following NICE grading*):

“(++) All or most of the checklist criteria have been fulfilled, where they have not been fulfilled the conclusions are very unlikely to alter. (+) Some of the checklist criteria have been fulfilled, where they have not been fulfilled, or not adequately described, the conclusions are unlikely to alter. (−) Few or no checklist criteria have been fulfilled and the conclusions are likely or very likely to alter.” (NICE 2012)

pp: “Indicates that for that particular aspect of study design, the study has been designed or conducted in such a way as to minimise the risk of bias”

p: “Indicates that either the answer to the checklist question is not clear from the way the study is reported, or that the study may not have addressed all potential sources of bias for that particular aspect of study design”

m: “Should be reserved for those aspects of the study design in which significant sources of bias may persist”

NR (not reported): “Should be reserved for those aspects in which the study under review fails to report how they have (or might have) been considered”

NA (not applicable): “Should be reserved for those study design aspects that are not applicable given the study design under review (for example, allocation concealment would not be applicable for case-control studies)”

(NICE 2012)

* IN focused on a special topic

^a^Data for age, sex and sample size only for participants affected by DM

Thirteen studies investigated IN as a primary outcome (primary research aim). Twelve of these  studies analysed the type of information needed by people with DM [3, 8, 9, 16–25], and one study investigated the relationships between IN, diagnosis and disease [4] (Table 1). The other studies (*n* = 12) reported IN as a secondary outcome and focused predominantly on other topics, e.g. information exchange, patient experience and information sources [6, 26–36]. Only two studies addressed unmet IN [3, 17], although these were often mentioned in the discussion of the publications [3, 8, 17, 24, 36]. The quality of information provided, in terms of readability and comprehensiveness, was not analysed in the included studies.

Altogether, we identified 14 qualitative studies, six quantitative studies and five mixed-method studies. Four different methods were reported throughout: interviews [3, 4, 8, 9, 17, 19, 23], group methods [16, 20, 24], surveys using written questionnaires [3, 21, 25], and website evaluation [18, 22]. Most of the instruments were specifically developed for the respective study, and five were validated regarding their understandability, suitability and feasibility [3, 4, 8, 9, 21, 23]. Validation regarding the validity and reliability of the instruments was not reported.

Eighteen studies used ʻopen questionsʼ (e.g. ‘Can you give an example of the type of information you have searched for?’) [4, 6, 8, 9, 16, 17, 19, 20, 23–31, 33, 36], while four studies used ʻclosed questionsʼ [3, 8, 9, 21, 25]. Two of the included studies analysed online posts [18, 32], and one examined e-mail requests [22], whereas 12 of them asked for IN focusing on one special topic, e.g. ‘pregnancy planning and childbearing’ [21, 22, 29], ‘information on medication’ [4, 16, 17], ‘oral health’ [28], ‘fitness and nutrition’ [30, 33], ‘quality of diabetes care’ [36], ‘automatic telephone outreach’ [26] and ‘blood pressure control’ [6]. Some studies used more than one approach.

The critical appraisal showed that three of the 25 identified studies met all or most of the NICE checklist criteria. The other studies fulfilled some (*n* = 14) or a few criteria (*n* = 8). It was noticeable that within the qualitative studies, most of the criteria were fulfilled but eight of 14 studies did not describe the role of the researcher sufficiently, and six gave no indications concerning ethical approval. None of the included quantitative studies reported how selection bias was minimised, and included studies using mixed-method design reported little about the quantitative part of their study design. The results of the critical appraisals are shown in Table 1.

### Content of information needs

The content analysis (Table 2) identified nine main types of IN of people with DM and 28 subtypes. The main categories are ‘treatment-process’, ‘course of disease’, ‘abnormalities of glucose metabolism’, ‘diabetes through the life cycle’, ‘pathophysiology of diabetes’, ‘research’, ‘coping’, ‘support’, and ‘prevention’. IN on the ‘treatment process’ were reported most frequently throughout the studies, in particular, ‘medication’ (*n* = 12), ‘diabetes self-management’ (*n* = 11) and ‘nutrition’ (*n* = 11). IN regarding the ‘course of disease’ were the second highest reported, particularly ‘consequences of diabetes’ (*n* = 16), e.g. consequences concerning physical health, lifestyle and social life. Only four studies reported IN on ‘coping’ and ‘support’, and two reported on ‘prevention’.

**Table 2:** Categories of information needs

Main categories	Definition	Sub-categories	Study designs		
			Qualitative studies	Quantitative studies	Mixed-method studies
Treatment process [3, 8, 9, 16–21, 23–25] [26, 27, 30–33, 36]*	IN concerning administration or application of remedies to a patient, or concerning a disease or injury as well as medicinal or surgical management; therapy	Medication	[16–20, 25]	[23, 24]; [32]*	[3, 8, 9, 21]
		Diabetes self-management	[18–20, 25]; [26, 27, 31]*	[23]; [32]*	[8, 9, 21]
		Nutrition	[19]; [26, 30, 31, 36]*	[23]; [32]*	[3, 8, 21]; [33]*
		Foot care		[24]	[8, 21]
		Treatment options			[3, 9]
		Emergency management	[19, 20]		
		Monitoring	[36]*	[24]	
Course of disease [3, 8, 9, 18, 19, 21–25] [6, 26, 28, 31–33, 35, 36]*	All information needs on topics related to the course of disease (progression through a development or period of illness)	Consequences (e.g. lifestyle, social life, physical health)	[18, 19, 25]; [26, 28, 31, 36]*	[23, 24]; [32, 35]*	[3, 8, 9, 21]; [6, 33]*
		Symptoms and diagnosis	[22]; [36]*	[23]	[9]
		Cure	[19, 22]; [26]*		
		Information on condition			[33]*
		Prognosis	[26]*		
Abnormalities of glucose metabolism [3, 4, 8, 19, 22–25] [27, 33]*	Information needs related to defined types of diseases with abnormal glucose metabolism (impaired glucose tolerance and impaired fasting glycaemia [37])	T1DM and T2DM	[19, 20, 25]	[23, 24]	[33]*
		Unspecified type of diabetes		[4]	[3, 8]
		MODY	[22]		
		GDM	[27]*		[33]*
Diabetes through the life cycle [20–22, 25]; [29, 32]*	Information needs related to topics that are part of different stages of the human life course—traditionally this includes childhood, adolescence, adulthood and old age	Reproduction (e.g. birth control, fertility, pregnancy)	[20, 22, 25]; [29]*	[32]*	[21]
		Puberty	[18]		
		Climacteric	[20]		
Pathophysiology of diabetes [9, 19, 21, 22, 24, 25]	Diabetes-related information needs on topics that refer to the pathophysiology of diabetes (e.g. impaired insulin secretion and increased insulin resistance [38])	Aetiology of diabetes	[19]		[9, 21]
		Pathogenesis of diabetes	[19]		
		Genetics	[22, 25]		
		Blood glucose levels		[24]	
Research [19, 21, 22]; [32, 34, 35]*	Information needs related to research, defined as current scientific knowledge, or studies, on diabetes	State of research	[19, 22]	[32, 34, 35]*	[21]
Coping [18, 19, 21, 23]	“Action regulation under stress including the ways that people mobilize, guide, manage, energize, and direct behaviour, emotion, and orientation, or how they fail to do so” under stressful conditions [39]	Diabetes-related family conflicts	[18, 19]		[21]
		Stress management		[23]	
Support [18, 24]; [32, 34]*	Information needs related to support that is defined as a person or thing that provides aid or assistance in managing diabetes	Medical support and financial support	[18]	[24, 32, 34]*	
Prevention [8, 9, 20]	Information needs related to prevention that is defined in terms of interventions that are provided before the initial onset of the diabetes	Not specified	[20]		[8, 9]

*IN: secondary outcome

### Information needs in subgroups and factors associated to IN

Specific comparisons between subgroups or analysis of associated factors using, for instance, regression models were performed minimally in the identified studies. Only one mixed-method study made a comparison between type 1 DM (T1DM) and type 2 DM (T2DM) [3]. Hence, we could only try to find subgroup-specific IN from studies that addressed certain groups as people with T1DM or T2DM or women with gestational DM (GDM). Only two studies investigated related factors such as, for example, socio-economic status [4, 8, 9]. Duggan et al. was the only study that performed quantitative statistics. The authors found, for example, that higher socio-economic status was positively correlated with the need for drug information [4]. More complex factors such as concepts like participation preferences or seeking behaviour were analysed more frequently than often-investigated associated factors such as age and sex, and these analyses were predominantly performed in qualitative studies, in particular in the study by St. Jean [8, 9] and Whetstone [23]. In the following, we describe the main findings.

### Information needs and types of diabetes

Twelve studies (13 publications) explicitly addressed people with T2DM [8, 9, 16, 17, 20, 23, 24, 28, 30, 31, 33, 34, 36] and six explicitly addressed people with T1DM [6, 18, 19, 21, 29, 32]. Additionally, four studies addressed different types of DM [3, 22, 25, 35], and two did not specify the type of DM [4, 26]. Finally, two studies focussed on women with GDM [22, 27] (Table 3). Only one mixed-method study made a comparison between T1DM and T2DM and showed that contents of patient-identified concerns between these subgroups are similar, apart from ʻnot knowing enoughʼ (T1DM) and ʻconcerns about futureʼ (T2DM) [3].

**Table 3:** Identified categories of IN by subgroups of people with DM

Categories	T1DM adults	T1DM children, adolescents	T2DM (including taking oral diabetes medication)	GDM	MODY	Unspecified type of diabetes
	(*N* = 7)	(*N* = 3)	(*N* = 14)^#^	(*N* = 2)	(*N* = 1)	(*N* = 5)^$^
Treatment process	[3, 21, 25] [32]*	[18, 19]	[3, 8, 9, 16, 17, 20, 23–25] [30, 31, 33, 34, 36]*	[27]*		[3] [26]*
Course of disease	[3, 21, 25] [6, 32]*	[18, 19]	[3, 8, 9, 20, 23–25] [28, 31, 33, 36]*		[22]	[26, 35]*
Abnormalities of glucose metabolism		[19]	[8, 20, 23–25] [33]*	[27]*	[22]	[3, 4]
Diabetes through the life cycle	[21, 22, 25] [29, 32]*	[18, 22]	[20]	[22]		[22]
Pathophysiology of diabetes		[19]	[9, 24]			
Research	[21, 22] [32]*	[19]	[34]*			[35]*
Coping	[3, 21]	[18, 19];	[9, 23]			
Support	[32]*		[34]*			[3]
Prevention			[8, 9, 20]			

#Twelve studies including IN exclusively of people with T2DM [8, 9, 16, 17, 20, 23, 24, 28, 30, 31, 33, 34, 36], 2 studies including in inter alia people with T2DM [3, 25]

$Three studies where the type of DM of the included population is not defined [22, 26, 35], 2 studies including IN that cannot be assigned certainly to a defined type of DM [3, 4]

*IN: secondary outcome

Overall, no striking differences between the IN of people with different types of DM were identified or noticeable in one certain group. Almost all the studies, with the exception of studies involving people with GDM and maturity onset diabetes of the young (MODY) [22, 27], reported IN in the categories ‘treatment process’, ‘course of disease’, ‘pathophysiology of diabetes’, ‘research’, ‘coping’, ‘support’ and ‘prevention’. With the exception of the studies involving people with T1DM, all the studies reported IN regarding ‘abnormalities of glucose metabolism’. Furthermore, ‘diabetes through the life cycle’ was addressed in most of the studies, but not in the study including people with MODY.

### Information needs and age

Overall, only a few differences of IN between people with DM in different age groups were identified in the studies; however, some were reported. Young people with T1DM were particularly interested in ‘diabetes through the life cycle’, e.g. ‘pregnancy’. There were two populations of young people identified: children with mean age 10 to 13 [19] and adolescents and young adults aged between 14 and 25 [18, 22]. Several IN were reported by both groups; however, there were also differences: the study by Olsen Roper et al. reporting IN of children between 10 and 13 years old showed that the population of children was particularly interested in the topic ‘course of disease’, especially ‘cure’ issues [19]. Additionally, they were interested in ‘abnormalities of glucose metabolism’, particularly ‘pathogenesis’ and ‘aetiology’ of diabetes [19]. In contrast, the population of adolescents and young adults was interested in ‘diabetes through the life cycle’, particularly in ‘puberty’ [18], ‘family founding’ [22] and ‘pregnancy’ [22].

Older people with DM were interested in ‘treatment process’, the ‘course of disease’ and the ‘abnormalities of glucose metabolism’. A further differentiation in age groups, e.g. ‘elderly’ and ‘very old’, cannot be derived from the studies.

### Information needs and information provision as well as information seeking

Six studies addressed IN in association with information provision and seeking [8, 9, 16, 18, 21–23]. General information about oral anti-hyperglycaemic medication and diabetes is provided preferably by general practitioners [16]. Furthermore, the provision of Internet-based information was recommended for the needs of childbearing women and young women, respectively [21, 22]. Information seeking in forums was associated with IN on ‘social support’, ‘life tasks’, ‘factual information’ and ‘management information’ [18]. Material items (such as ‘books, news clippings, journal articles, printouts from an Internet site or notes of references that are maintained in the home’) are associated with different IN [23]. For example, IN on the topic ‘nutrition’ are associated with information sources such as cookbooks, hand-outs and self-selected website print-outs [23]. Generally, more frequent seeking for diabetes-related information was associated with lower ratings for the usefulness of information regarding diabetes-related complications [8, 9].

### Information and participation preferences, knowledge about and experience with diabetes

Three studies addressed IN in association with participation preferences. All three studies analysed IN in relation to decision-making and involvement in the decision process [3, 8, 9, 25]. St. Jean pointed out that an involvement in decision-making is related to higher ratings for the usefulness of information [8, 9].

Two studies addressed IN in association with ʻknowledgeʼ and ʻ(feelings about) diabetes experienceʼ [8, 9, 19]. Knowledge, diabetes experience and IN are strongly related. Therefore, both studies recommended identifying the individual level of knowledge and whether this is correct [8, 9, 19]. Furthermore, clear, optimistic, less-uncertain feelings, or support in diabetes experience were associated with different IN, e.g. ‘diabetes management’, ‘causes of diabetes’, ‘diabetes-related complications’ [8, 9].

### Stage of the disease

One study that focused on people with DM who had recently started treatment with oral anti-hyperglycaemic drugs addressed IN in association with the stage of the disease. It pointed out that people with DM who recently started treatment with anti-hyperglycaemic drugs are in need of diabetes medication information such as drug-related issues [16].

## Discussion

This is the first systematic review of studies dealing with IN of people with DM. We identified 25 studies (26 publications). This is a limited number compared with, say, cancer, where a large number of quantitative and qualitative IN studies (*n* = 112) already exist [37]. This is surprising, since it is known that people with DM have a higher or similarly high need for information compared with people with other chronic diseases [3, 4]. Looking for the content of IN, it was comparable to those found in people with cancer, such as ʻprognosis of diseaseʼ, ʻdiagnostic testsʼ, ʻtreatmentʼ, ʻself-careʼ, and ʻemotional and psychological needsʼ [37]. However, Duggan et al. found that people with DM have a higher need for information about drugs than people with cancer or cardiovascular disease [4].

It would be interesting to look for differences between people with diabetes and cancer or other chronic diseases in more detail.

Besides the low number of studies, it became obvious that differences between patient groups such as male and female patients, different age groups or types of diabetes have not been analysed so far. Factors associated with IN are rarely investigated. In cases in which analysis was performed, mainly the more complex factors such as participation preferences or information seeking were investigated, and it was particularly done in qualitative studies, which were highly heterogeneous. Also, changes in IN during the course of the disease are poorly investigated, although they may be expected.

Instruments for collecting IN data from individuals with DM have not been validated. Similar results were identified in cancer studies and showed that only a minority of instruments for the collection of IN are validated [37].

### Implications for research

There is a need for research on several levels. First, compared with other chronic diseases, there is a huge lack of studies addressing IN of people with DM, although DM is one of the largest public health issues [38]. Due to the low number of studies dealing with unmet needs, and considering the relevance of this topic mentioned in the studies, there is a need for further research. Second, differences between the types of DM populations including gender aspects and patient characteristics should be analysed. Third, there is a need for research to show associations of variables with IN and to amend the findings from qualitative studies via qualitative analyses. Available knowledge about the IN and associated factors and concepts can be used in targeted counselling and to strengthen the health literacy of people with DM. Finally, methods and instruments should be further developed against a theoretical background and validated.

### Limitations

We conducted a comprehensive and sensitive search that was also pre-tested. The study selection and critical appraisal were performed by two reviewers. Two other reviewers checked all the decisions. The critical appraisals were performed for each study design. However, there were still difficulties to provide a clear decision based on the reports.

The identified studies have several limitations: only three qualitative studies met all the quality criteria. None of the quantitative studies met all the criteria. The comparability of the IN categories is restricted because the IN were collected by different study designs. Some of them were collected in the context of a specific health-related topic or by using different methods or instruments.

## Conclusion

There is a limited number of studies analysing IN in DM, and there is a low number of studies investigating differences between subgroups of DM populations, including gender aspects or changes of information needs during the disease. This should be further investigated.

### Additional file

Additional file 1: PRISMA (Preferred Reporting Items for Systematic review and Meta-Analysis) Checklist 2009. Checklist: recommended items to address in a systematic review. (DOC 145 kb)

## Appendix 1

### Search terms (26/06/2014) [11]

#### MEDLINE (OVID)

Database: MEDLINE (1946–2014)

Research period: unlimited

**Table:**

Search step	Hits	Search
1	702	EXP INFORMATION SEEKING BEHAVIOR/
2	1718	EXP INFORMATION LITERACY/
3	3397	EXP CONSUMER HEALTH INFORMATION/
4	70,372	EXP PATIENT EDUCATION AS TOPIC/
5	600	EXP HEALTH COMMUNICATION/
6	1279	1 OR 5
7	34	6 AND (diabetes OR diabetic OR niddm OR iddm OR t2 dm OR t1 dm OR prediabetes OR prediabetic OR pre-diabetes OR pre-diabetic OR impaired glucose).ti,ab.
8	3488	2 OR 3
9	80	8 AND (diabetes OR diabetic OR niddm OR iddm OR t2 dm OR t1 dm OR prediabetes OR prediabetic OR pre-diabetes OR pre-diabetic OR impaired glucose).ti,ab. AND information.ti,ab.
10	107	4 AND ((diabetes OR diabetic OR niddm OR iddm OR t2 dm OR t1 dm OR prediabetes OR prediabetic OR pre-diabetes OR pre-diabetic OR impaired glucose).ti,ab. AND ((interest* OR need OR needs OR question* OR asking OR ask OR seek* OR search* OR demand OR desire OR request* OR call OR requirement OR requiring OR preference* OR wish OR wishes OR provision* OR expectation*) ADJ5 information)).ti,ab.
11	45	(information needs AND (diabetes OR diabetic OR niddm OR iddm OR t2 dm OR t1 dm OR prediabetes OR prediabetic OR pre-diabetes OR pre-diabetic OR impaired glucose)).ti,ab.
12	3935	((education OR communication) ADJ2 need OR needs OR preference*)).ti,ab.
13	85	12 AND (diabetes OR diabetic OR prediabetes OR prediabetic OR pre-diabetes OR pre-diabetic OR impaired glucose).ti.
14	1721	*PATIENT PREFERENCE/
15	13	14 AND (information OR education OR communication).ti,ab. AND (diabetes OR diabetic OR prediabetes OR prediabetic OR pre-diabetes OR pre-diabetic OR impaired glucose).ti,ab.
16	9730	((patient OR patient-centered) ADJ1 (need OR needs OR seek* OR search* OR demand OR desire OR preference* OR wish OR wishes OR provision* OR expectation*)).ti,ab.
17	34	16 AND (diabetes OR diabetic OR prediabetes OR prediabetic OR pre-diabetes OR pre-diabetic OR impaired glucose).ti. AND (need OR needs OR preference*).ti.
18	2	14 AND (facilitat* OR barrier* OR pitfall*).ti. AND (diabetes OR diabetic OR prediabetes OR prediabetic OR pre-diabetes OR pre-diabetic OR impaired glucose).ti,ab.
19	941	(((information OR education OR communication) ADJ3 (interest* OR need OR needs OR question* OR asking OR ask OR seek* OR search* OR demand OR desire OR request* OR call OR requirement OR requiring OR preference* OR wish OR wishes OR provision* OR expectation*)) AND (diabetes OR diabetic OR prediabetes OR prediabetic OR pre-diabetes OR pre-diabetic OR impaired glucose)).ti,ab.
20	178	19 AND (interest* OR need* OR question* OR ask* OR talk* OR online communi* OR message* OR seek* OR search* OR demand OR desire OR request* OR call OR requir* OR preference* OR wish* OR perception* OR provision* OR expectation* OR facilitat* OR barrier* OR pitfall*).ti.
21	4014	(information AND (interest* OR need OR needs OR question* OR asking OR ask OR seek* OR search* OR demand OR desire OR request* OR call OR requirement OR requiring OR preference*)).ti.
22	53	21 AND (diabetes OR diabetic OR prediabetes OR prediabetic OR pre-diabetes OR pre-diabetic OR impaired glucose).ti,ab.
23	5	((identify* ADJ2 (interest* OR need OR needs OR requirement OR preference*)) AND (diabetes OR diabetic OR prediabetes OR prediabetic OR pre-diabetes OR pre-diabetic OR impaired glucose)).ti.
24	19	(support* AND (interest* OR need OR needs OR requirement OR preference*)).ti. AND (diabetes OR diabetic OR prediabetes OR prediabetic OR pre-diabetes OR pre-diabetic OR impaired glucose).ti,ab.
25	497	7 OR 9 OR 10 OR 11 OR 13 OR 15 OR 17 OR 18 OR 20 OR 22 OR 23 OR 24

#### EMBASE (OVID)

Database: EMBASE (1974–2014)

Research period: unlimited

**Table:**

Search step	Hits	Search
1	989	EXP INFORMATION SEEKING/
2	187	EXP INFORMATION LITERACY/
3	2226	EXP CONSUMER HEALTH INFORMATION/
4	23,953	*PATIENT EDUCATION/
5	7326	*MEDICAL INFORMATION/
6	72	1 AND 5
7	2	6 AND (diabetes OR diabetic OR prediabetes OR prediabetic OR pre-diabetes OR pre-diabetic OR impaired glucose).ti,ab.
8	1164	1 OR 2
9	20	8 AND (diabetes OR diabetic OR prediabetes OR prediabetic OR pre-diabetes OR pre-diabetic OR impaired glucose).ti,ab.
10	36	3 AND (diabetes OR diabetic OR prediabetes OR prediabetic OR pre-diabetes OR pre-diabetic OR impaired glucose).ti,ab. AND (interest* OR need OR needs OR question* OR asking OR ask OR seek* OR search* OR demand OR desire OR request* OR call OR requirement OR requiring OR preference* OR wish OR wishes OR provision* OR expectation*).ti,ab.
11	65	5 AND (diabetes OR diabetic OR prediabetes OR prediabetic OR pre-diabetes OR pre-diabetic OR impaired glucose).ti,ab. AND (interest* OR need OR needs OR question* OR asking OR ask OR seek* OR search* OR demand OR desire OR request* OR call OR requirement OR requiring OR preference* OR wish OR wishes OR provision* OR expectation*).ti,ab.
12	100	10 OR 11
13	24	12 AND ((information OR education OR support) ADJ2 (interest* OR need OR needs OR question* OR asking OR ask OR seek* OR search* OR demand OR desire OR request* OR call OR requirement OR requiring OR preference* OR wish OR wishes OR provision* OR expectation*)).ti,ab.
14	43	4 AND ((diabetes OR diabetic OR niddm OR iddm OR t2 dm OR t1 dm OR prediabetes OR prediabetic OR pre-diabetes OR pre-diabetic OR impaired glucose).ti,ab. AND ((interest* OR need OR needs OR question* OR asking OR ask OR seek* OR search* OR demand OR desire OR request* OR call OR requirement OR requiring OR preference* OR wish OR wishes OR provision* OR expectation*) ADJ5 information)).ti,ab.
15	65	(information needs AND (diabetes OR diabetic OR niddm OR iddm OR t2 dm OR t1 dm OR prediabetes OR prediabetic OR pre-diabetes OR pre-diabetic OR impaired glucose)).ti,ab.
16	4941	((education OR communication) ADJ2 (need OR needs OR preference*)).ti,ab.
17	127	16 AND (diabetes OR diabetic OR prediabetes OR prediabetic OR pre-diabetes OR pre-diabetic OR impaired glucose).ti.
18	1354	*PATIENT PREFERENCE/
19	13	18 AND (information OR education OR communication).ti,ab. AND (diabetes OR diabetic OR prediabetes OR prediabetic OR pre-diabetes OR pre-diabetic OR impaired glucose).ti,ab.
20	13,294	((patient OR patient-centered) ADJ1 (need OR needs OR seek* OR search* OR demand OR desire OR preference* OR wish OR wishes OR provision* OR expectation*)).ti,ab.
21	52	20 AND (diabetes OR diabetic OR prediabetes OR prediabetic OR pre-diabetes OR pre-diabetic OR impaired glucose).ti. AND (need OR needs OR preference*).ti.
22	2	18 AND (facilitat* OR barrier* OR pitfall*).ti. AND (diabetes OR diabetic OR prediabetes OR prediabetic OR pre-diabetes OR pre-diabetic OR impaired glucose).ti,ab.
23	4637	(information AND (interest* OR need OR needs OR question* OR asking OR ask OR seek* OR search* OR demand OR desire OR request* OR call OR requirement OR requiring OR preference*)).ti.
24	66	23 AND (diabetes OR diabetic OR prediabetes OR prediabetic OR pre-diabetes OR pre-diabetic OR impaired glucose).ti,ab.
25	6	((identify* ADJ2 (interest* OR need OR needs OR requirement OR preference*)) AND (diabetes OR diabetic OR prediabetes OR prediabetic OR pre-diabetes OR pre-diabetic OR impaired glucose)).ti.
26	32	(support* AND (interest* OR need OR needs OR requirement OR preference*)).ti. AND (diabetes OR diabetic OR prediabetes OR prediabetic OR pre-diabetes OR pre-diabetic OR impaired glucose).ti,ab.
27	855	(((information OR education OR communication) ADJ2 (interest* OR need OR needs OR question* OR asking OR ask OR seek* OR search* OR demand OR desire OR request* OR call OR requirement OR requiring OR preference* OR wish OR wishes OR provision* OR expectation*)) AND (diabetes OR diabetic OR prediabetes OR prediabetic OR pre-diabetes OR pre-diabetic OR impaired glucose)).ti,ab.
28	158	27 AND (interest* OR need* OR question* OR ask* OR talk* OR online communi* OR message* OR seek* OR search* OR demand OR desire OR request* OR call OR requir* OR preference* OR wish* OR perception* OR provision* OR expectation* OR facilitat* OR barrier* OR pitfall*).ti.
29	440	7 OR 9 OR 13 OR 14 OR 15 OR 17 OR 19 OR 21 OR 22 OR 24 OR 25 OR 26 OR 28

#### PsycINFO, Journals@OVID (OVID)

Databases: PsycINFO (1806–2014), Journals@OVID

Research period: unlimited

**Table:**

Search step	Hits	Search
1	3002	EXP INFORMATION SEEKING/
2	15	1 AND (diabetes OR diabetic OR prediabetes OR prediabetic OR pre-diabetes OR pre-diabetic OR impaired glucose).ti,ab.
3	3027	CLIENT EDUCATION/
4	7	3 AND (diabetes OR diabetic OR prediabetes OR prediabetic OR pre-diabetes OR pre-diabetic OR impaired glucose).ti,ab. AND ((interest* OR need OR needs OR question* OR asking OR ask OR seek* OR search* OR demand OR desire OR request* OR call OR requirement OR requiring OR preference* OR wish OR wishes OR provision* OR expectation*) ADJ5 information).ti,ab.
5	28	(information needs AND (diabetes OR diabetic OR niddm OR iddm OR t2 dm OR t1 dm OR prediabetes OR prediabetic OR pre-diabetes OR pre-diabetic OR impaired glucose)).ti,ab.
6	5992	((education OR communication) ADJ2 (need OR needs OR preference*)).ti,ab.
7	91	6 AND (diabetes OR diabetic OR prediabetes OR prediabetic OR pre-diabetes OR pre-diabetic OR impaired glucose).ti.
8	7387	((patient OR patient-centered) ADJ1 (need OR needs OR seek* OR search* OR demand OR desire OR preference* OR wish OR wishes OR provision* OR expectation*)).ti,ab.
9	26	8 AND (diabetes OR diabetic OR prediabetes OR prediabetic OR pre-diabetes OR pre-diabetic OR impaired glucose).ti. AND (need OR needs OR preference*).ti.
10	4494	(information AND (interest* OR need OR needs OR question* OR asking OR ask OR seek* OR search* OR demand OR desire OR request* OR call OR requirement OR requiring OR preference*)).ti.
11	30	10 AND (diabetes OR diabetic OR prediabetes OR prediabetic OR pre-diabetes OR pre-diabetic OR impaired glucose).ti,ab.
12	9	((identify* ADJ2 (interest* OR need OR needs OR requirement OR preference*)) AND (diabetes OR diabetic OR prediabetes OR prediabetic OR pre-diabetes OR pre-diabetic OR impaired glucose)).ti.
13	29	(support* AND (interest* OR need OR needs OR requirement OR preference*)).ti. AND (diabetes OR diabetic OR prediabetes OR prediabetic OR pre-diabetes OR pre-diabetic OR impaired glucose).ti,ab.
14	509	(((information OR education OR communication) ADJ2 (interest* OR need OR needs OR question* OR asking OR ask OR seek* OR search* OR demand OR desire OR request* OR call OR requirement OR requiring OR preference* OR wish OR wishes OR provision* OR expectation*)) AND (diabetes OR diabetic OR prediabetes OR prediabetic OR pre-diabetes OR pre-diabetic OR impaired glucose)).ti,ab.
15	123	14 AND (interest* OR need* OR question* OR ask* OR talk* OR online communi* OR message* OR seek* OR search* OR demand OR desire OR request* OR call OR requir* OR preference* OR wish* OR perception* OR provision* OR expectation* OR facilitat* OR barrier* OR pitfall*).ti.
16	259	2 OR 4 OR 5 OR 7 OR 9 OR 11 OR 12 OR 13 OR 15

#### CINAHL (EBSCO)

Database: CINAHL (1981–2014)

Research period: unlimited

**Table:**

Search step	Hits	Search
S1	198	MW INFORMATION NEEDS AND (diabetes OR diabetic OR niddm OR iddm OR t2 dm OR t1 dm OR prediabetes OR prediabetic OR pre-diabetes OR pre-diabetic OR impaired glucose)
S2	8	TI information needs AND (diabetes OR diabetic OR niddm OR iddm OR t2 dm OR t1 dm OR prediabetes OR prediabetic OR pre-diabetes OR pre-diabetic OR impaired glucose)
S3	57	TI (education OR communication) AND TI (need OR needs OR preference*) AND (diabetes OR diabetic OR niddm OR iddm OR t2 dm OR t1 dm OR prediabetes OR prediabetic OR pre-diabetes OR pre-diabetic OR impaired glucose)
S4	103	TI (patient OR patient-centered) AND TI (need* OR seek* OR talk* OR online communi* OR message OR search* OR demand OR desire OR preference* OR wish* OR provision* OR perception* OR expectation*) AND (diabetes OR diabetic OR niddm OR iddm OR t2 dm OR t1 dm OR prediabetes OR prediabetic OR pre-diabetes OR pre-diabetic OR impaired glucose)
S5	20	TI information AND TI (interest* OR need OR needs OR question* OR asking OR ask OR seek* OR search* OR demand OR desire OR request* OR call OR requirement OR requiring OR preference*) AND (diabetes OR diabetic OR niddm OR iddm OR t2 dm OR t1 dm OR prediabetes OR prediabetic OR pre-diabetes OR pre-diabetic OR impaired glucose)
S6	352	S1 OR S2 OR S3 OR S4 OR S5

#### The Cochrane Library (Wiley)

Databases:

Cochrane Database of Systematic Reviews (1996–2014), Database of Abstracts of Reviews of Effects (DARE) (1994–2014), Cochrane Central Register of Controlled Trials (1898–2014), Cochrane Methodology Register (1904–2012), Health Technology Assessment (HTA) (1989–2014), NHS Economic Evaluation Database (NHS EED) (1968–2014)

Research period: unlimited

**Table:**

Search step	Hits	Search
#1	0	(information need* AND (diabetes OR diabetic OR prediabetes OR prediabetic OR pre-diabetes OR pre-diabetic OR impaired glucose)):ti
#2	4	((education OR communication OR information) AND (need OR needs OR preference*) AND (diabetes OR diabetic OR niddm OR iddm OR t2 dm OR t1 dm OR prediabetes OR prediabetic OR pre-diabetes OR pre-diabetic OR impaired glucose)):ti
#3	8	((information* OR knowledge) AND (need OR needs OR needed OR seek* OR talk* OR online communi* OR search* OR demand OR desire OR preference* OR wish* OR provision* OR perception* OR expectation*) AND (diabetes OR diabetic OR niddm OR iddm OR t2dm OR t1dm OR prediabetes OR prediabetic OR pre-diabetes OR pre-diabetic OR impaired glucose)):ti
#4	3	(information AND (interest* OR need OR needs OR question* OR asking OR ask OR seek* OR search* OR demand OR desire OR request* OR call OR requirement OR requiring OR preference*) AND (diabetes OR diabetic OR niddm OR iddm OR t2dm OR t1dm OR prediabetes OR prediabetic OR pre-diabetes OR pre-diabetic OR impaired glucose)):ti
#5	32	((need OR needs OR seek* OR search* OR demand OR desire OR preference* OR wish OR wishes OR provision* OR expectation*) AND (diabetes OR diabetic OR prediabetes OR prediabetic OR pre-diabetes OR pre-diabetic OR impaired glucose) AND (information OR education OR support)):ti
#6	224	(information need AND (diabetes OR diabetic OR prediabetes OR prediabetic OR pre-diabetes OR pre-diabetic OR impaired glucose)):ti,ab,kw
#7	12	#6 AND (information OR knowledge):ti
#5	41	#1 OR #2 OR #3 OR #4 OR #5 OR #7 Cochrane Reviews:1 Other Reviews:14 Trials:12 Methods Studies:Methods Studie4 Technology Assessments:0 Economic Evaluations:10 Cochrane Groups:0

#### Web of Science (Thomson Reuters)

Database: Web of Science (1950–2014)

Research period: unlimited

**Table:**

Search step	Hits	Search
1	123	TITLE: (information AND (interest* OR need OR needs OR question* OR talk* OR ask* OR online communi* OR seek* OR search* OR demand OR desire OR request* OR call OR requir* OR perception* OR preference*) AND (diabetes OR diabetic OR prediabetes OR prediabetic OR pre-diabetes OR pre-diabetic OR impaired glucose)) OR TITLE: ((need OR needs OR seek* OR search* OR demand OR desire OR preference* OR wish OR wishes OR provision* OR expectation*) AND (diabetes OR diabetic OR prediabetes OR prediabetic OR pre-diabetes OR pre-diabetic OR impaired glucose) AND (information OR education OR support OR perception*)) OR TITLE: ((education OR communication) AND (need OR needs OR preference*) AND (diabetes OR diabetic OR prediabetes OR prediabetic OR pre-diabetes OR pre-diabetic OR impaired glucose)) Timespan = All Years Lemmatization = On

#### ERIC (Institute of Education Sciences)

Database: ERIC (1966–2014)

Research period: unlimited

**Table:**

Search step	Hits	Search
1	6	"INFORMATION NEED*" AND "DIABETES"

#### ScienceDirect (Elsevier)

Database: ScienceDirect (Elsevier, Segmente ‘Decision Sciences, Medicine’, ‘Medicine and Dentistry’, ‘Neuroscience’, ‘Nursing and Health professions’, ‘Pharmacology, Toxicology and Pharmaceutical Science’, ‘Psychology’, ‘Social Sciences’) (1823–2014)

Research period: unlimited

**Table:**

Search step	Hits	Search
1	9	TITLE((information) AND (diabetes OR diabetic)) AND TITLE-ABSTR-KEY((interest* OR need OR needs OR question* OR ask* OR talk* OR online communi* OR seek* OR search* OR demand OR desire OR request* OR call OR requir* OR perception* OR preference*))

#### CCMed, Deutsches Ärzteblatt (DIMDI)

Database: CCMed (2001–2014), Deutsches Ärzteblatt (1996–2014)

Research period: unlimited

**Table:**

Search step	Hits	Search
1	3	FT = (diabetes; diabetic; pre-diabet?; prediabet?; impaired glucose) AND TI = (information?; education; knowledge)) AND FT = (interest?; need?; question?; talk?; online communi?; ask?; seek?; search?; demand; desire; request?; call; requir?; perception?; preference?)
2	9	FT = (diabetes; diabetic; pre-diabet?; prediabet?; impaired glucose) AND FT = informationsbedarf
3	12	1 OR 2

#### Karlsruher virtueller Katalog (Karlsruher Institut für Technologie)

Database: Karlsruher virtueller Katalog (DIMDI)

Research period: unlimited

**Table:**

Search step	Hits^1^	Search
1	0	information need* AND (diabet* OR pre-diabet* OR prediabet*)
2	0	informationsbedarf AND (diabet* OR pre-diabet* OR prediabet*)
3	0	preferences AND (diabet* OR pre-diabet* OR prediabet*)

^1^After screening

## Appendix 2

### Search terms (Update 26/06/2015) [11]

#### MEDLINE (OVID)

Database: MEDLINE (1946–2015)

Research period: 10/06/2014–01/07/2015

**Table:**

Search step	Hits	Search
1	896	EXP INFORMATION SEEKING BEHAVIOR/
2	2260	EXP INFORMATION LITERACY/
3	4241	EXP CONSUMER HEALTH INFORMATION/
4	72,616	EXP PATIENT EDUCATION AS TOPIC/
5	782	EXP HEALTH COMMUNICATION/
6	1652	1 OR 5
7	35	6 AND (diabetes OR diabetic OR niddm OR iddm OR t2 dm OR t1 dm OR prediabetes OR prediabetic OR pre-diabetes OR pre-diabetic OR impaired glucose).ti,ab.
8	4349	2 OR 3
9	87	8 AND (diabetes OR diabetic OR niddm OR iddm OR t2 dm OR t1 dm OR prediabetes OR prediabetic OR pre-diabetes OR pre-diabetic OR impaired glucose).ti,ab. AND information.ti,ab.
10	108	4 AND ((diabetes OR diabetic OR niddm OR iddm OR t2 dm OR t1 dm OR prediabetes OR prediabetic OR pre-diabetes OR pre-diabetic OR impaired glucose).ti,ab. AND ((interest* OR need OR needs OR question* OR asking OR ask OR seek* OR search* OR demand OR desire OR request* OR call OR requirement OR requiring OR preference* OR wish OR wishes OR provision* OR expectation*) ADJ5 information)).ti,ab.
11	47	(information needs AND (diabetes OR diabetic OR niddm OR iddm OR t2 dm OR t1 dm OR prediabetes OR prediabetic OR pre-diabetes OR pre-diabetic OR impaired glucose)).ti,ab.
12	4253	((education OR communication) ADJ2 (need OR needs OR preference*)).ti,ab.
13	88	12 AND (diabetes OR diabetic OR prediabetes OR prediabetic OR pre-diabetes OR pre-diabetic OR impaired glucose).ti.
14	2210	*PATIENT PREFERENCE/
15	16	14 AND (information OR education OR communication).ti,ab. AND (diabetes OR diabetic OR prediabetes OR prediabetic OR pre-diabetes OR pre-diabetic OR impaired glucose).ti,ab.
16	10,638	((patient OR patient-centered) ADJ1 (need OR needs OR seek* OR search* OR demand OR desire OR preference* OR wish OR wishes OR provision* OR expectation*)).ti,ab.
17	43	16 AND (diabetes OR diabetic OR prediabetes OR prediabetic OR pre-diabetes OR pre-diabetic OR impaired glucose).ti. AND (need OR needs OR preference*).ti.
18	3	14 AND (facilitat* OR barrier* OR pitfall*).ti. AND (diabetes OR diabetic OR prediabetes OR prediabetic OR pre-diabetes OR pre-diabetic OR impaired glucose).ti,ab.
19	1051	(((information OR education OR communication) ADJ3 (interest* OR need OR needs OR question* OR asking OR ask OR seek* OR search* OR demand OR desire OR request* OR call OR requirement OR requiring OR preference* OR wish OR wishes OR provision* OR expectation*)) AND (diabetes OR diabetic OR prediabetes OR prediabetic OR pre-diabetes OR pre-diabetic OR impaired glucose)).ti,ab.
20	194	19 AND (interest* OR need* OR question* OR ask* OR talk* OR online communi* OR message* OR seek* OR search* OR demand OR desire OR request* OR call OR requir* OR preference* OR wish* OR perception* OR provision* OR expectation* OR facilitat* OR barrier* OR pitfall*).ti.
21	4256	(information AND (interest* OR need OR needs OR question* OR asking OR ask OR seek* OR search* OR demand OR desire OR request* OR call OR requirement OR requiring OR preference*)).ti.
22	57	21 AND (diabetes OR diabetic OR prediabetes OR prediabetic OR pre-diabetes OR pre-diabetic OR impaired glucose).ti,ab.
23	5	((identify* ADJ2 (interest* OR need OR needs OR requirement OR preference*)) AND (diabetes OR diabetic OR prediabetes OR prediabetic OR pre-diabetes OR pre-diabetic OR impaired glucose)).ti.
24	22	(support* AND (interest* OR need OR needs OR requirement OR preference*)).ti. AND (diabetes OR diabetic OR prediabetes OR prediabetic OR pre-diabetes OR pre-diabetic OR impaired glucose).ti,ab.
25	541	7 OR 9 OR 10 OR 11 OR 13 OR 15 OR 17 OR 18 OR 20 OR 22 OR 23 OR 24
26	50	limit 25 to ed="20140610-20150701"

#### EMBASE (OVID)

Database: EMBASE (1974–2015)

Research period: 24. CW 2014–27. CW 2015

**Table:**

Search step	Hits	Search
1	1371	EXP INFORMATION SEEKING/
2	238	EXP INFORMATION LITERACY/
3	2647	EXP CONSUMER HEALTH INFORMATION/
4	25,038	*PATIENT EDUCATION/
5	7879	*MEDICAL INFORMATION/
6	102	1 AND 5
7	2	6 AND (diabetes OR diabetic OR prediabetes OR prediabetic OR pre-diabetes OR pre-diabetic OR impaired glucose).ti,ab.
8	1595	1 OR 2
9	29	8 AND (diabetes OR diabetic OR prediabetes OR prediabetic OR pre-diabetes OR pre-diabetic OR impaired glucose).ti,ab.
10	40	3 AND (diabetes OR diabetic OR prediabetes OR prediabetic OR pre-diabetes OR pre-diabetic OR impaired glucose).ti,ab. AND (interest* OR need OR needs OR question* OR asking OR ask OR seek* OR search* OR demand OR desire OR request* OR call OR requirement OR requiring OR preference* OR wish OR wishes OR provision* OR expectation*).ti,ab.
11	69	5 AND (diabetes OR diabetic OR prediabetes OR prediabetic OR pre-diabetes OR pre-diabetic OR impaired glucose).ti,ab. AND (interest* OR need OR needs OR question* OR asking OR ask OR seek* OR search* OR demand OR desire OR request* OR call OR requirement OR requiring OR preference* OR wish OR wishes OR provision* OR expectation*).ti,ab.
12	108	10 OR 11
13	25	12 AND ((information OR education OR support) ADJ2 (interest* OR need OR needs OR question* OR asking OR ask OR seek* OR search* OR demand OR desire OR request* OR call OR requirement OR requiring OR preference* OR wish OR wishes OR provision* OR expectation*)).ti,ab.
14	44	4 AND ((diabetes OR diabetic OR niddm OR iddm OR t2dm OR t1dm OR prediabetes OR prediabetic OR pre-diabetes OR pre-diabetic OR impaired glucose).ti,ab. AND ((interest* OR need OR needs OR question* OR asking OR ask OR seek* OR search* OR demand OR desire OR request* OR call OR requirement OR requiring OR preference* OR wish OR wishes OR provision* OR expectation*) ADJ5 information)).ti,ab.
15	69	(information needs AND (diabetes OR diabetic OR niddm OR iddm OR t2dm OR t1dm OR prediabetes OR prediabetic OR pre-diabetes OR pre-diabetic OR impaired glucose)).ti,ab.
16	5502	((education OR communication) ADJ2 (need OR needs OR preference*)).ti,ab.
17	149	16 AND (diabetes OR diabetic OR prediabetes OR prediabetic OR pre-diabetes OR pre-diabetic OR impaired glucose).ti.
18	1877	*PATIENT PREFERENCE/
19	19	18 AND (information OR education OR communication).ti,ab. AND (diabetes OR diabetic OR prediabetes OR prediabetic OR pre-diabetes OR pre-diabetic OR impaired glucose).ti,ab.
20	15113	((patient OR patient-centered) ADJ1 (need OR needs OR seek* OR search* OR demand OR desire OR preference* OR wish OR wishes OR provision* OR expectation*)).ti,ab.
21	64	20 AND (diabetes OR diabetic OR prediabetes OR prediabetic OR pre-diabetes OR pre-diabetic OR impaired glucose).ti. AND (need OR needs OR preference*).ti.
22	2	18 AND (facilitat* OR barrier* OR pitfall*).ti. AND (diabetes OR diabetic OR prediabetes OR prediabetic OR pre-diabetes OR pre-diabetic OR impaired glucose).ti,ab.
23	5002	(information AND (interest* OR need OR needs OR question* OR asking OR ask OR seek* OR search* OR demand OR desire OR request* OR call OR requirement OR requiring OR preference*)).ti.
24	71	23 AND (diabetes OR diabetic OR prediabetes OR prediabetic OR pre-diabetes OR pre-diabetic OR impaired glucose).ti,ab.
25	6	((identify* ADJ2 (interest* OR need OR needs OR requirement OR preference*)) AND (diabetes OR diabetic OR prediabetes OR prediabetic OR pre-diabetes OR pre-diabetic OR impaired glucose)).ti.
26	42	(support* AND (interest* OR need OR needs OR requirement OR preference*)).ti. AND (diabetes OR diabetic OR prediabetes OR prediabetic OR pre-diabetes OR pre-diabetic OR impaired glucose).ti,ab.
27	984	(((information OR education OR communication) ADJ2 (interest* OR need OR needs OR question* OR asking OR ask OR seek* OR search* OR demand OR desire OR request* OR call OR requirement OR requiring OR preference* OR wish OR wishes OR provision* OR expectation*)) AND (diabetes OR diabetic OR prediabetes OR prediabetic OR pre-diabetes OR pre-diabetic OR impaired glucose)).ti,ab.
28	175	27 AND (interest* OR need* OR question* OR ask* OR talk* OR online communi* OR message* OR seek* OR search* OR demand OR desire OR request* OR call OR requir* OR preference* OR wish* OR perception* OR provision* OR expectation* OR facilitat* OR barrier* OR pitfall*).ti.
29	510	7 OR 9 OR 13 OR 14 OR 15 OR 17 OR 19 OR 21 OR 22 OR 24 OR 25 OR 26 OR 28
30	67	limit 29 to em="201424-201527"

#### PsycINFO, Journals@OVID (OVID)

Database: PsycINFO (1806–2015) Journals@OVID

Research period: 10/06/2014–01/07/2015 (PsycINFO) and publication year 2014–2015 (Journals@Ovid)

**Table:**

Search step	Hits	Search
1	3175	EXP INFORMATION SEEKING/
2	19	1 AND (diabetes OR diabetic OR prediabetes OR prediabetic OR pre-diabetes OR pre-diabetic OR impaired glucose).ti,ab.
3	3209	CLIENT EDUCATION/
4	10	3 AND (diabetes OR diabetic OR prediabetes OR prediabetic OR pre-diabetes OR pre-diabetic OR impaired glucose).ti,ab. AND ((interest* OR need OR needs OR question* OR asking OR ask OR seek* OR search* OR demand OR desire OR request* OR call OR requirement OR requiring OR preference* OR wish OR wishes OR provision* OR expectation*) ADJ5 information).ti,ab.
5	30	(information needs AND (diabetes OR diabetic OR niddm OR iddm OR t2dm OR t1dm OR prediabetes OR prediabetic OR pre-diabetes OR pre-diabetic OR impaired glucose)).ti,ab.
6	6616	((education OR communication) ADJ2 (need OR needs OR preference*)).ti,ab.
7	100	6 AND (diabetes OR diabetic OR prediabetes OR prediabetic OR pre-diabetes OR pre-diabetic OR impaired glucose).ti.
8	8087	((patient OR patient-centered) ADJ1 (need OR needs OR seek* OR search* OR demand OR desire OR preference* OR wish OR wishes OR provision* OR expectation*)).ti,ab.
9	29	8 AND (diabetes OR diabetic OR prediabetes OR prediabetic OR pre-diabetes OR pre-diabetic OR impaired glucose).ti. AND (need OR needs OR preference*).ti.
10	4835	(information AND (interest* OR need OR needs OR question* OR asking OR ask OR seek* OR search* OR demand OR desire OR request* OR call OR requirement OR requiring OR preference*)).ti.
11	35	10 AND (diabetes OR diabetic OR prediabetes OR prediabetic OR pre-diabetes OR pre-diabetic OR impaired glucose).ti,ab.
12	9	((identify* ADJ2 (interest* OR need OR needs OR requirement OR preference*)) AND (diabetes OR diabetic OR prediabetes OR prediabetic OR pre-diabetes OR pre-diabetic OR impaired glucose)).ti.
13	38	(support* AND (interest* OR need OR needs OR requirement OR preference*)).ti. AND (diabetes OR diabetic OR prediabetes OR prediabetic OR pre-diabetes OR pre-diabetic OR impaired glucose).ti,ab.
14	572	(((information OR education OR communication) ADJ2 (interest* OR need OR needs OR question* OR asking OR ask OR seek* OR search* OR demand OR desire OR request* OR call OR requirement OR requiring OR preference* OR wish OR wishes OR provision* OR expectation*)) AND (diabetes OR diabetic OR prediabetes OR prediabetic OR pre-diabetes OR pre-diabetic OR impaired glucose)).ti,ab.
15	136	14 AND (interest* OR need* OR question* OR ask* OR talk* OR online communi* OR message* OR seek* OR search* OR demand OR desire OR request* OR call OR requir* OR preference* OR wish* OR perception* OR provision* OR expectation* OR facilitat* OR barrier* OR pitfall*).ti.
16	294	2 OR 4 OR 5 OR 7 OR 9 OR 11 OR 12 OR 13 OR 15
17	210	limit 16 to up="20140610-20150701"
18	209	remove duplicates from 17
19	57	limit 18 to yr="2013 - 2015" Journals@Ovid: 43 PsycInfo: 14

#### CINAHL (EBSCO)

Database: CINAHL (1981–2015)

Research period: 01/06/2014–31/07/2015

**Table:**

Search step	Hits	Search
S1	206	MW INFORMATION NEEDS AND (diabetes OR diabetic OR niddm OR iddm OR t2 dm OR t1 dm OR prediabetes OR prediabetic OR pre-diabetes OR pre-diabetic OR impaired glucose)
S2	7	TI information needs AND (diabetes OR diabetic OR niddm OR iddm OR t2 dm OR t1 dm OR prediabetes OR prediabetic OR pre-diabetes OR pre-diabetic OR impaired glucose)
S3	51	TI (education OR communication) AND TI (need OR needs OR preference*) AND (diabetes OR diabetic OR niddm OR iddm OR t2 dm OR t1 dm OR prediabetes OR prediabetic OR pre-diabetes OR pre-diabetic OR impaired glucose)
S4	110	TI (patient OR patient-centered) AND TI (need* OR seek* OR talk* OR online communi* OR message OR search* OR demand OR desire OR preference* OR wish* OR provision* OR perception* OR expectation*) AND (diabetes OR diabetic OR niddm OR iddm OR t2 dm OR t1 dm OR prediabetes OR prediabetic OR pre-diabetes OR pre-diabetic OR impaired glucose)
S5	22	TI information AND TI (interest* OR need OR needs OR question* OR asking OR ask OR seek* OR search* OR demand OR desire OR request* OR call OR requirement OR requiring OR preference*) AND (diabetes OR diabetic OR niddm OR iddm OR t2 dm OR t1 dm OR prediabetes OR prediabetic OR pre-diabetes OR pre-diabetic OR impaired glucose)
S6	372	S1 OR S2 OR S3 OR S4 OR S5
S7	21	(EM 20140601-20150731) AND S6

#### The Cochrane Library (Wiley)

Databases: Cochrane Database of Systematic Reviews (1996–2015), Database of Abstracts of Reviews of Effects (DARE) (1994–2015), Cochrane Central Register of Controlled Trials (1898–2015), Cochrane Methodology Register (1904–2012), Health Technology Assessment (HTA) (1989–2015), NHS Economic Evaluation Database (NHS EED) (1968–2015)

Research period: 06/2014–06/2015

**Table:**

Search step	Hits	Search
#1	0	(information need* AND (diabetes OR diabetic OR prediabetes OR prediabetic OR pre-diabetes OR pre-diabetic OR impaired glucose)):ti
#2	4	((education OR communication OR information) AND (need OR needs OR preference*) AND (diabetes OR diabetic OR niddm OR iddm OR t2 dm OR t1 dm OR prediabetes OR prediabetic OR pre-diabetes OR pre-diabetic OR impaired glucose)):ti
#3	8	((information* OR knowledge) AND (need OR needs OR needed OR seek* OR talk* OR online communi* OR search* OR demand OR desire OR preference* OR wish* OR provision* OR perception* OR expectation*) AND (diabetes OR diabetic OR niddm OR iddm OR t2 dm OR t1 dm OR prediabetes OR prediabetic OR pre-diabetes OR pre-diabetic OR impaired glucose)):ti
#4	3	(information AND (interest* OR need OR needs OR question* OR asking OR ask OR seek* OR search* OR demand OR desire OR request* OR call OR requirement OR requiring OR preference*) AND (diabetes OR diabetic OR niddm OR iddm OR t2 dm OR t1 dm OR prediabetes OR prediabetic OR pre-diabetes OR pre-diabetic OR impaired glucose)):ti
#5	40	((need OR needs OR seek* OR search* OR demand OR desire OR preference* OR wish OR wishes OR provision* OR expectation*) AND (diabetes OR diabetic OR prediabetes OR prediabetic OR pre-diabetes OR pre-diabetic OR impaired glucose) AND (information OR education OR support)):ti
#6	117	(information need AND (diabetes OR diabetic OR prediabetes OR prediabetic OR pre-diabetes OR pre-diabetic OR impaired glucose)):ti,ab,kw
#7	7	#6 AND (information OR knowledge):ti
#5	46	#1 OR #2 OR #3 OR #4 OR #5 OR #7 Cochrane Reviews:1 Other Reviews:20 Trials:10 Methods Studies:2 Technology Assessments:0 Economic Evaluations:13 Cochrane Groups:0
#6	0	#1 OR #2 OR #3 OR #4 OR #5 OR #7Online Publication Date in the last 12 months

#### Web of Science (Thomson Reuters)

Database: Web of Science (1950–2015)

Research period: publication year 2014–2015

**Table:**

Search step	Hits	Search
1	163	TITLE: (information AND (interest* OR need OR needs OR question* OR talk* OR ask* OR online communi* OR seek* OR search* OR demand OR desire OR request* OR call OR requir* OR perception* OR preference*) AND (diabetes OR diabetic OR prediabetes OR prediabetic OR pre-diabetes OR pre-diabetic OR impaired glucose)) OR TITLE: ((need OR needs OR seek* OR search* OR demand OR desire OR preference* OR wish OR wishes OR provision* OR expectation*) AND (diabetes OR diabetic OR prediabetes OR prediabetic OR pre-diabetes OR pre-diabetic OR impaired glucose) AND (information OR education OR support OR perception*)) OR TITLE: ((education OR communication) AND (need OR needs OR preference*) AND (diabetes OR diabetic OR prediabetes OR prediabetic OR pre-diabetes OR pre-diabetic OR impaired glucose)) Timespan = All Years Lemmatization = On
2	26	TITLE: ((information AND (interest* OR need OR needs OR question* OR talk* OR ask* OR online communi* OR seek* OR search* OR demand OR desire OR request* OR call OR requir* OR perception* OR preference*) AND (diabetes OR diabetic OR prediabetes OR prediabetic OR pre-diabetes OR pre-diabetic OR impaired glucose))) OR TITLE: (((need OR needs OR seek* OR search* OR demand OR desire OR preference* OR wish OR wishes OR provision* OR expectation*) AND (diabetes OR diabetic OR prediabetes OR prediabetic OR pre-diabetes OR pre-diabetic OR impaired glucose) AND (information OR education OR support OR perception*))) OR TITLE: (((education OR communication) AND (need OR needs OR preference*) AND (diabetes OR diabetic OR prediabetes OR prediabetic OR pre-diabetes OR pre-diabetic OR impaired glucose))) Refined by: PUBLICATION YEARS: (2015 OR 2014) Timespan = All years Search language = Auto

#### ERIC (Institute of Education Sciences)

Database: ERIC (1966–2015)

Research period: unlimited.

**Table:**

Search step	Hits	Search
1	7	“INFORMATION NEED*” AND “DIABETES”

Download: 1 data set

#### ScienceDirect (Elsevier)

Database: ScienceDirect (Elsevier, Segmente ‘Decision Sciences, Medicine’, ‘Medicine and Dentistry’, ‘Neuroscience’, ‘Nursing and Health professions’, ‘Pharmacology, Toxicology and Pharmaceutical Science’, ‘Psychology’, ‘Social Sciences’) (1823–2015)

Research period: Publication year 2014–2015

**Table:**

Search step	Hits	Search
1	11	TITLE((information) AND (diabetes OR diabetic)) AND TITLE-ABSTR-KEY((interest* OR need OR needs OR question* OR ask* OR talk* OR online communi* OR seek* OR search* OR demand OR desire OR request* OR call OR requir* OR perception* OR preference*))
2	4	pub-date > 2013 and TITLE((information) AND (diabetes OR diabetic)) AND TITLE-ABSTR-KEY((interest* OR need OR needs OR question* OR ask* OR talk* OR online communi* OR seek* OR search* OR demand OR desire OR request* OR call OR requir* OR perception* OR preference*))

#### CCMed (MedPilot, ZBMed)

Database: CCMed (2001–2015)

Research period: Publication year 2014–2015

**Table:**

Search step	Hits	Search
1	37	TI = ((diabetes OR diabetic OR pre-diabet* OR prediabet* OR impaired glucose) AND (interest* OR need* OR question* OR talk* OR online communi* OR ask* OR seek* OR search* OR demand OR desire OR request* OR call OR requir* OR perception* OR preference*)) AND TI = ((information* OR education OR knowledge)) Jahre 2014–2015: 1
2	392	TI = ((diabetes OR diabetic OR pre-diabet* OR prediabet* OR impaired glucose)) AND TI = ((information* OR education OR knowledge)) Jahre 2014–2015: 21

Download: 22 datasets

#### Deutsches Ärzteblatt (Deutscher Ärzteverlag)

Database: Deutsches Ärzteblatt (1996–2015)

Research period: Publication year 2014–2015

**Table:**

Search step	Hits	Search
1	0	Diabetes informationsbedarf Publicationyear 2014–2015
2	0	Diabetes information need Publicationyear 2014–2015

#### Karlsruher virtueller Katalog (Karlsruher Institut für Technologie)

Database: Karlsruher virtueller Katalog (DIMDI)

Research period: unlimited

**Table:**

Search step	Hits^1^	Search
1	0	information need* AND (diabet* OR pre-diabet* OR prediabet*)
2	0	informationsbedarf AND (diabet* OR pre-diabet* OR prediabet*)
3	0	preferences AND (diabet* OR pre-diabet* OR prediabet*)

^1^After screening

## Abbreviations

A.I.: Andrea Icks
A.S.: Astrid Stephan
DM: Diabetes mellitus
GDM: gestational diabetes
IN: Information needs
J.G.: Jutta Genz
L.B.: Lisa Biernatzki
M.R.: Michaela Ritschel
MODY: Maturity-onset diabetes of the young
S.D.: Sigrid Droste
S.K.: Silke Kuske
T1DM: Type 1 diabetes mellitus
T2DM: Type 2 diabetes mellitus
