# Correction to: Information needs in people with diabetes mellitus: a systematic review

**DOI:** 10.1186/s13643-019-1072-y

**Published:** 2019-07-02

**Authors:** Lisa Biernatzki, Silke Kuske, Jutta Genz, Michaela Ritschel, Astrid Stephan, Christina Bächle, Sigrid Droste, Sandra Grobosch, Nicole Ernstmann, Nadja Chernyak, Andrea Icks

**Affiliations:** 1 Institute for Health Services Research and Health Economics, Centre for Health and Society, Faculty of Medicine, Heinrich Heine University, Moorenstraße 5, 40225 Düsseldorf, Germany; 2 Institute for Health Services Research and Health Economics, German Diabetes Center (DDZ), Leibniz Institute for Diabetes Research, at Heinrich Heine University Düsseldorf, Auf’m Hennekamp 65, 40225 Düsseldorf, Germany; 3 Center for Health Communication and Health Services Research (CHSR), Department for Psychosomatic Medicine and Psychotherapy, University Hospital Bonn, Sigmund-Freud-Str. 25, 53127 Bonn, Germany; 4 German Center for Diabetes Research (DZD), Ingolstädter Landstraße 1, 85764 Neuherberg, Germany

**Correction to: Syst Rev**

**https://doi.org/10.1186/s13643-018-0690-0**

Following publication of the original article [1], the authors opted to revise Table 1. Below is the updated version of the table.

**Table 1:** Overview – included studies

Author/year	Methods	Sample size^(a)^	Population characteristics			Outcomes	Aims / Findings	Associated factors	Critical appraisal	Number of criteria
			DM type	Sex^(a)^	Age^(a)^					
In (Primary Outcome)										
Qualitative Studies										
Lamberts et al. 2010 [15]*	Focus group	*n* = 42	Type 2 DM (T2DM)	f (*n* = 13), m (*n* = 19)	18–80	Information needed and provided according to patients starting oral T2DM medication	The study explored the information needs of patients who have recently started treatment with oral antidiabetics and analysed the provision of information. The study showed that patients are in need of diabetes-medication information such as drug-related issues.	x	+	11/12
Lee et al. 2007 [16]*	Interview	*n* = 24	T2DM	f (*n* = 6), m (*n* = 18)	44–79	Knowledge of diabetes and prescribed medicines; experiences with medicines information; consumer-specific written medicines information needs; written leaflets on medicine information needs	A ‘Consumer Involvement Cycle’ is developed to assist researchers, and to analyse perspectives and needs on medicines information to develop written medicines information. The study identified a lack of written medicine information for people with diabetes, who wish for specific information about their medicine (mechanisms of action, administering instructions, drug-related issues)		+	11/12
Olsen Roper et al. 2009 [18]	Interview	*n* = 58	Type 1 DM (T1DM)	f (*n* = 37), m (*n* = 21)	8–18	Existing knowledge about diabetes and information needs about diabetes	This study explored information needs of children with DMT1 among other things concerning diabetes care, its pathophysiology, consequences, treatment and possible cure.	x	+	11/12
Ravert et al. 2004 [17]	Website evaluation	n = NR (*n* = 340 Messages)	T1DM	f (79%), m (21%) (sex was identified in 48,5% of all messages)	11–19	Reasons for posts; topics of requests; reliability and coding issues; forum differences; gender differences; age and duration of illness	This study explored messages posted on public web-based forums. The people with diabetes expressed information needs regarding the consequences of the disease, social support and life tasks.	x	+	10/12
Savage et al. 2009 [19]	Focus group	*n* = 13	T2DM	f (*n* = 9), m (*n* = 4)	26–44	Preferred content and delivery mode of education and information	This study showed that the main information need of people with DMT2 between the ages of 25 to 45 was related to adequate information on managing diabetes themselves, e.g., medication, preventing diabetes, pregnancy and emergency management not sufficiently covered at present.	x	+	11/12
Van Esch et al. 2010 [21]	Website evaluation	*n* = 77 (*n* = 158Messages)	T1DM, T2DM, Gestational DM (GDM), Maturity onset diabetes of the young (MODY)/Not defined	f, m (no number available for people with DM)	< 20–> 41	Information needs of online consumers (genetics and diabetes)	This study identified information needs about the role of inheritance in diabetes.	x	_	7/12
Wilson 2013 [24]	Questionnaire	*n* = 30	T1DM, T2DM	f (*n* = 16), m (*n* = 14)	22–64	Access; information type;	The study explored the method preferred by people with diabetes to access information about their condition, and what type of information they require.	x	_	1/12
Quantitative Studies										
Duggan et al. 2008 [4]*	Interview	*n* = 117	not defined (and other diseases)	f, m (no number available for peole with DM)	55.5–60.3	Relationships between information needs, diagnosis and disease	The study showed that different diagnoses and diseases are associated with different medicine information needs.		+	2PP, 4P, 4 M, 0NR, 9NA
Whitford et al. 2013 [23]	Focus groups and semistructured interviews (within a randomised controlled trial)	*n* = 29 (support groups)	T2DM	NR	NR	Information needs of participants with T2DM	This explored the use of a system of patient-generated “frequently asked questions” in order to gain insight into the information needs of participants.		_	1PP, 3P, 4 M, 1NR, 10NA
Whetstone 2014 [22]	Interview	*n* = 21	T2DM	f (*n* = 15), m (*n* = 6)	38–79	Kept health information and information needs	This study explored information behaviour and information needs.	x	+	3PP, 3P, 4 M, 1NR, 8NA
Mixed-Method Studies										
Beeney et al. 1996 [3]	Interview + questionnaire	*n* = 1145	T1DM, T2DM	f (*n* = 573), m (*n* = 572)	39.9 ± 19;64.2 ± 12	Information needs and emotional support	They study explored patient information needs for emotional support and information preferences.	x	_	5/21 (11 NA)
Sparud-Lundin et al. 2011 [20]*	Questionnaire	*n* = 105	T1DM	f (*n* = 105)	≤30–36≥	Socio demographic factors; use of the internet (information seeking and communication); diabetes-related issues and specific questions on needs in relation to childbearing; Expectations of web-based support	This study explored the internet use, the needs and expectations of web-based information and communication. Information needs were expressed regarding diabetes-related aspects, regarding e.g., pregnancy, childbirth, and parenthood.	x	_	5/21 (9 NA)
St. Jean 2012 [8], 2014 [9]	Interview, questionnaire, card sorting, timeline	*n* = 34	T2DM	f (*n* = 20), m (*n* = 14)	30-89	Changes across the time: information seeking and use; awareness and capability of articulating information needs; usefulness of sources and types of diabetes-related information	This study explored information behaviour and its changes across time, and identified different content types of diabetes-related information needs, e.g., risk factors, medication, exercise.	x	+	7 of 21 (13 NA)
In (Secondary Outcome)										
Qualitative Studies										
Goldman et al. 2008 [25]*	Interview	*n* = 36	not defined	f (*n* = 22), m (*n* = 14)	20–61+	Patients’ opinions about automated speech-recognition telephone technology	While developing an automated telephone outreach intervention for people with diabetes, the study obtained IN of patients on nutrition and dietary advice, consequences and blood glucose control.		+	10/12
Hjelm et al. 2008 [26]*	Interview	*n* = 23	GDM	f (*n* = 23)	23–41	Beliefs about health and health care	While exploring beliefs about health, illness and healthcare in women with Gestational Diabetes Mellitus (GDM), further study results identified IN about GDM and its treatment.		++	12/12
Lindenmeyer et al. 2013 [27]*	Interview	*n* = 20	T2DM	f (*n* = 8), m (*n* = 12)	40–82	[27] awareness; interaction with dental health professionals and information exchange; information preferences	The study explored the awareness of people with type 2 diabetes, how they communicate with dentists and professionals (primary care), and preferences of how to receive care and information related to oral health.		+	11/12
McCorry et al. 2012 [28]*	Interview	*n* = 14	T1DM	f (*n* = 14)	21–38	Attitude toward pregnancy planning and pre-conception	The study explored attitudes toward pregnancy planning and antenatal care. IN of women with T1DM concerning antenatal care, pregnancy and diabetes management in this time.		++	12/12
Meyfroidt et al. 2013 [29]*	Focus group	*n* = 21	T2DM	f (*n* = 7), m (*n* = 14)	41–85	Use of information sources; information seeking; problems encountered by the patients	While obtaining data to determine how people with diabetes seek and use information sources for their diet, further results identified IN concerning food characteristics.	x	++	12/12
Peel et al. 2004 [30]	Interview	*n* = 40	T2DM	f (*n* = 19), m (*n* = 21)	21–77	‘Suspected diabetes’ route to diagnosis; ‘illness’ route to diagnosis; ‘routine’ route to diagnosis; information provision at diagnosis; overall emotional reactions to diagnosis	During research on patients’ views on information provision at the time of diagnosis, the study identified the need for information on course of disease and its consequences, diabetes management and advice on nutrition.		+	9/12
Wilkinson et al. 2014 [35] *	Interview	*n* = 47	T2DM	f (*n* = 22), m (*n* = 25)	34–85	Diagnosis of diabetes; symptoms; access; experience of diabetes services; current health; self-management/support	The study explored the quality of diabetes care and identified IN on, for example, diet, risk and complications explained.		_	6/12
Quantitative Studies										
Chen et al. 2012 [31]	Web based blog analysis	*n* = 516 (*n* = 2806 Messages)	T1DM	NR	NR	Patient experience (emotional, temporal)	The study explored online discussion forums for three conditions: breast cancer, T1DM and fibromyalgia. It showed that many people with T1DM addressed topics of diabetes management. However, they were also interested in website references, sharing experiences and support.		_	0PP, 1P, 8 M, 0NR, 10NA
Hajos et al. 2011 [33]	Questionnaire	*n* = 1609	T2DM	f (*n* = 660), m (*n* = 949)	51.4 ± 12.5	Seriousness of their diabetes, diabetes-related distress, worries about complications, need for care improvement	The study explored the extent to which physicians understand T2DM; e.g. patients’ perceptions of seriousness and emotional distress, and needs for care improvement. The study showed that people need more information about treatment options, where to get support and the newest information.		+	1PP, 7P, 1 M, 0NR, 9NA
Robertson et al. 2005 [34]	Questionnaire	*n* = 70	T1DM, T2DM	f (*n* = 27), m (*n* = 43)	16–79	Sources and adequacy of information	This study explored the sources of information and their adequacy for supplying diabetes information. The people with diabetes expressed a lack of information about their condition.	x	+	1PP, 5P. 3 M, 1NR, 9NA
Mixed-Method Studies										
Frandsen et al. 2002 [32]	Interview + questionnaire	*n* = 123	T2DM	f (*n* = 59), m (*n* = 64)	45–60	Issues and barriers relating to patient compliance	The study explored issues and barriers relating to patient compliance and showed that the people with T2DM want more information about their condition.		_	0/21 (19 NA)
Mühlhauser et al. 1988 [6]*	Interview + questionnaire	*n* = 37	T1DM	f (*n* = 13), m (*n* = 24)	38 ± 9	Blood pressure control (compliance)	The study explored the degree of blood pressure control and identified the need for more information about high blood pressure.		+	4/21 (9 NA)

* IN focused on a special topic; (a) Data for age, sex and sample size only for participants affected by DM Quantitative or qualitative studies, mixed-method studies (following NICE grading): “(++) All or most of the checklist criteria have been fulfilled, where they have not been fulfilled the conclusions are very unlikely to alter. (+) Some of the checklist criteria have been fulfilled, where they have not been fulfilled, or not adequately described, the conclusions are unlikely to alter. (−) Few or no checklist criteria have been fulfilled and the conclusions are likely or very likely to alter.” (NICE 2012)

pp: “Indicates that for that particular aspect of study design, the study has been designed or conducted in such a way as to minimize the risk of bias”

p: “Indicates that either the answer to the checklist question is not clear from the way the study is reported, or that the study may not have addressed all potential sources of bias for that particular aspect of study design”

m: “Should be reserved for those aspects of the study design in which significant sources of bias may persist”

NR (not reported): “Should be reserved for those aspects in which the study under review fails to report how they have (or might have) been considered”

NA (not applicable): “Should be reserved for those study design aspects that are not applicable given the study design under review (for example, allocation concealment would not be applicable for case-control studies)”

(NICE 2012)
