# Supplementary material for: Information needs in people with diabetes mellitus: a systematic review
Source: Syst Rev. 2018 Feb 14;7:27. doi: 10.1186/s13643-018-0690-0 (PMC5813383; doi:10.1186/s13643-018-0690-0)
Supplement: Supplementary file 1 — PRISMA (Preferred Reporting Items for Systematic review and Meta-Analysis) Checklist 2009. Checklist: recommended items to address in a systematic review. (DOC 145 kb) [file 13643_2018_690_MOESM1_ESM.doc]

| **Section/topic** | **#** | **Checklist item** |  | **Reported on page #** |
| --- | --- | --- | --- | --- |
| **TITLE** | | |  |  |
| Title | 1 | Identify the report as a systematic review, meta-analysis, or both. | *Information needs in people with diabetes mellitus: a systematic review* | 1 |
| **ABSTRACT** | | |  |  |
| Structured summary | 2 | Provide a structured summary including, as applicable: background; objectives; data sources; study eligibility criteria, participants, and interventions; study appraisal and synthesis methods; results; limitations; conclusions and implications of key findings; systematic review registration number. | *Background: Diabetes as a chronical disease is one of the largest public health issues. People with diabetes have to perform self-management on their disease on a daily basis. For that knowledge on diabetes is necessary and in order to that, need-driven information.*  *Objectives: The purpose of this study was to identify and analyse currently available knowledge on information needs (IN) of people with diabetes mellitus (DM), also considering possible differences between subgroups and associated factors.*  *Data sources: MEDLINE, EMBASE, The Cochrane Library etc. were searched until June 2015.*  *Eligibility criteria, participants: Publications that addressed self-reported information needs of people with diabetes mellitus were included.*  *Study appraisal: Each study was assessed by using critical appraisal tools, e.g. from the UK National Institute for Health and Care Excellence.*  *Synthesis methods: For data-extraction a data-extraction-sheet following requirements of Cochrane Collaboration was used. Content analysis was performed systematically by developing categories inductively from the studies concerning diabetes-related IN.*  *Results: In total, 1,993 publications were identified and 26 were finally included. Nine main types of IN were identified, including ‘treatment-process’, ‘course of disease’, ‘abnormalities of glucose metabolism’ and ‘diabetes through the life cycle’. Six main-categories of associated factors and concepts were developed, e.g.: ‘information provision’, ‘change of information needs’, and ‘information and participation preferences’.*  *Limitations: A comprehensive and sensitive search was conducted and pre-tested. Study selection and critical appraisal were performed by two reviewers. Two other reviewers checked all the decisions. The critical appraisals were performed for each study design. However, there were still difficulties to provide a clear decision based on the reports.*  *Included studies have several limitations: only three qualitative studies and none of the quantitative studies, met all the quality criteria. The comparability of IN categories is restricted because IN were collected by different study designs. Some of them were collected in the context of a specific health-related topic or by using different methods or instruments.*  *Conclusion: Information needs in people with DM, appear to be high, yet poorly investigated. Research is needed regarding differences between diverse diabetes populations, including gender aspects or changes in IN during the disease course.*  *Implications of key findings: Available knowledge about the IN and associated factors and concepts can be used in targeted counselling, and to strengthen the health literacy of people with DM.*  *Review is not registered.* | 7-9, 21, 22 |
| **INTRODUCTION** | | |  |  |
| Rationale | 3 | Describe the rationale for the review in the context of what is already known. | *People with DM have to perform comprehensive self-management interventions to achieve good diabetes control [1]. In order to make adequate decisions concerning their illness, a sufficient level of disease-related information is required [2]. In fact, people with DM communicate a particularly high need for information, higher than people with cancer or cardiovascular diseases, for example [3, 4]. However, it seems people with DM do not feel adequately informed about their condition or regarding medication use [3].* | 7 |
| Objectives | 4 | Provide an explicit statement of questions being addressed with reference to participants, interventions, comparisons, outcomes, and study design (PICOS). | *This systematic review aims to identify and analyse currently available knowledge on the IN of people with DM, also considering possible differences between subgroups and associated factors.* | 7 |
| **METHODS** | | |  |  |
| Protocol and registration | 5 | Indicate if a review protocol exists, if and where it can be accessed (e.g., Web address), and, if available, provide registration information including registration number. | *In accordance with the guidelines, our systematic review protocol was registered with the International Prospective Register of Systematic Reviews (PROSPERO) on 27.11.2015 (CRD42015029610). It can be found on: http://www.crd.york.ac.uk/PROSPERO/display_record.asp?ID=CRD42015029610* | 9 |
| Eligibility criteria | 6 | Specify study characteristics (e.g., PICOS, length of follow-up) and report characteristics (e.g., years considered, language, publication status) used as criteria for eligibility, giving rationale. | *Studies, addressing self-reported IN of people with DM (any type), as primary or secondary research aim were included published from the inception of each database up to June 2015 with a German or English title and abstract, and full text in an language. Original qualitative, quantitative or mixed methods studies were included. Systematic and narrative reviews, meta-analyses and qualitative meta-syntheses were also included.*  *Studies reporting the IN of relatives or healthcare professionals were excluded, as well as studies where relatives or healthcare professionals reported IN of people with DM. Publications without available references, letters / short reports, abstracts, editorials, comments or discussion papers were excluded.* | 9-10 |
| Information sources | 7 | Describe all information sources (e.g., databases with dates of coverage, contact with study authors to identify additional studies) in the search and date last searched. | *Searched databases were: MEDLINE, EMBASE, CINAHL, ScienceDirect, the Cochrane Library, Web of Science, PsycINFO, CCMed, ERIC and Journals@OVID, Deutsches Ärzteblatt and Karlsruher virtueller Katalog.Publications. Publications were included that had been published from the inception of each database up to June 2015. Full texts were screened with the aim of identifying further original studies using backward citation tracking.* | 9 |
| Search | 8 | Present full electronic search strategy for at least one database, including any limits used, such that it could be repeated. | *Search strategie in MEDLINE (OVID) from 1946 to June 2014:*   | **Search step** | **Hits** | **Search** | | --- | --- | --- | | 1 | 702 | EXP INFORMATION SEEKING BEHAVIOR/ | | 2 | 1718 | EXP INFORMATION LITERACY/ | | 3 | 3397 | EXP CONSUMER HEALTH INFORMATION/ | | 4 | 70372 | EXP PATIENT EDUCATION AS TOPIC/ | | 5 | 600 | EXP HEALTH COMMUNICATION/ | | 6 | 1279 | 1 OR 5 | | 7 | 34 | 6 AND (diabetes OR diabetic OR niddm OR iddm OR t2dm OR t1dm OR prediabetes OR prediabetic OR pre-diabetes OR pre-diabetic OR impaired glucose).ti,ab. | | 8 | 3488 | 2 OR 3 | | 9 | 80 | 8 AND (diabetes OR diabetic OR niddm OR iddm OR t2dm OR t1dm OR prediabetes OR prediabetic OR pre-diabetes OR pre-diabetic OR impaired glucose).ti,ab. AND information.ti,ab. | | 10 | 107 | 4 AND ((diabetes OR diabetic OR niddm OR iddm OR t2dm OR t1dm OR prediabetes OR prediabetic OR pre-diabetes OR pre-diabetic OR impaired glucose).ti,ab. AND ((interest* OR need OR needs OR question* OR asking OR ask OR seek* OR search* OR demand OR desire OR request* OR call OR requirement OR requiring OR preference* OR wish OR wishes OR provision* OR expectation*) ADJ5 information)).ti,ab. | | 11 | 45 | (information needs AND (diabetes OR diabetic OR niddm OR iddm OR t2dm OR t1dm OR prediabetes OR prediabetic OR pre-diabetes OR pre-diabetic OR impaired glucose)).ti,ab. | | 12 | 3935 | ((education OR communication) ADJ2 need OR needs OR preference*)).ti,ab. | | 13 | 85 | 12 AND (diabetes OR diabetic OR prediabetes OR prediabetic OR pre-diabetes OR pre-diabetic OR impaired glucose).ti. | | 14 | 1721 | *PATIENT PREFERENCE/ | | 15 | 13 | 14 AND (information OR education OR communication).ti,ab. AND (diabetes OR diabetic OR prediabetes OR prediabetic OR pre-diabetes OR pre-diabetic OR impaired glucose).ti,ab. | | 16 | 9730 | ((patient OR patient-centered) ADJ1 (need OR needs OR seek* OR search* OR demand OR desire OR preference* OR wish OR wishes OR provision* OR expectation*)).ti,ab. | | 17 | 34 | 16 AND (diabetes OR diabetic OR prediabetes OR prediabetic OR pre-diabetes OR pre-diabetic OR impaired glucose).ti. AND (need OR needs OR preference*).ti. | | 18 | 2 | 14 AND (facilitat* OR barrier* OR pitfall*).ti. AND (diabetes OR diabetic OR prediabetes OR prediabetic OR pre-diabetes OR pre-diabetic OR impaired glucose).ti,ab. | | 19 | 941 | (((information OR education OR communication) ADJ3 (interest* OR need OR needs OR question* OR asking OR ask OR seek* OR search* OR demand OR desire OR request* OR call OR requirement OR requiring OR preference* OR wish OR wishes OR provision* OR expectation*)) AND (diabetes OR diabetic OR prediabetes OR prediabetic OR pre-diabetes OR pre-diabetic OR impaired glucose)).ti,ab. | | 20 | 178 | 19 AND (interest* OR need* OR question* OR ask* OR talk* OR online communi* OR message* OR seek* OR search* OR demand OR desire OR request* OR call OR requir* OR preference* OR wish* OR perception* OR provision* OR expectation* OR facilitat* OR barrier* OR pitfall*).ti. | | 21 | 4014 | (information AND (interest* OR need OR needs OR question* OR asking OR ask OR seek* OR search* OR demand OR desire OR request* OR call OR requirement OR requiring OR preference*)).ti. | | 22 | 53 | 21 AND (diabetes OR diabetic OR prediabetes OR prediabetic OR pre-diabetes OR pre-diabetic OR impaired glucose).ti,ab. | | 23 | 5 | ((identify* ADJ2 (interest* OR need OR needs OR requirement OR preference*)) AND (diabetes OR diabetic OR prediabetes OR prediabetic OR pre-diabetes OR pre-diabetic OR impaired glucose)).ti. | | 24 | 19 | (support* AND (interest* OR need OR needs OR requirement OR preference*)).ti. AND (diabetes OR diabetic OR prediabetes OR prediabetic OR pre-diabetes OR pre-diabetic OR impaired glucose).ti,ab. | | 25 | 497 | 7 OR 9 OR 10 OR 11 OR 13 OR 15 OR 17 OR 18 OR 20 OR 22 OR 23 OR 24 | | Appendix 1 |
| Study selection | 9 | State the process for selecting studies (i.e., screening, eligibility, included in systematic review, and, if applicable, included in the meta-analysis). | *Inclusion and exclusion criteria were pre-tested on 380 records and finally discussed. Then two reviewers independently selected the articles, first by title and abstract and thereafter by full text. All decisions were checked by two other reviewers. Unclear decisions were resolved by an additional reviewer.*  *Full texts were screened with the aim of identifying further original studies using backward citation tracking.* | 10 |
| Data collection process | 10 | Describe method of data extraction from reports (e.g., piloted forms, independently, in duplicate) and any processes for obtaining and confirming data from investigators. | *A data extraction sheet was developed following the requirements of Cochrane [5]. We extracted the type of information needed by people with DM, and, if investigated, by different subgroups, such as type of DM and age.* | 10 |
| Data items | 11 | List and define all variables for which data were sought (e.g., PICOS, funding sources) and any assumptions and simplifications made. | *The following information were extracted from the included studies:*  *author, date, methods, findings, result of the critical appraisal of the study quality. Furthermore, the main categories of IN were described, as well as those related to subgroups of people with DM, and the associated factors.* | 10, Table 1 |
| Risk of bias in individual studies | 12 | Describe methods used for assessing risk of bias of individual studies (including specification of whether this was done at the study or outcome level), and how this information is to be used in any data synthesis. | *Each study was critically appraised separately by using design-specific critical appraisal tools from the UK National Institute for Health and Care Excellence (NICE) [6].* *Mixed methods were analysed by the Mixed Methods Appraisal Tool (MMAT) – Version 2011 [7]* | 11 |
| Summary measures | 13 | State the principal summary measures (e.g., risk ratio, difference in means). | *Not applicable* | / |
| Synthesis of results | 14 | Describe the methods of handling data and combining results of studies, if done, including measures of consistency (e.g., I^2^) for each meta-analysis. | *A content analysis was conducted, developing categories according to the topics of the review questions, in particular to assess the reported and analysed types of information needed. Furthermore, IN-associated factors were extracted and analysed via a content analysis. Codings were developed inductively [8] using a coding protocol, and revised critically. the main categories of IN were described, as well as those related to subgroups of people with DM, and the associated factors.* | 10, 11 |

Page 1 of 2

| **Section/topic** | **#** | **Checklist item** |  | **Reported on page #** |
| --- | --- | --- | --- | --- |
| Risk of bias across studies | 15 | Specify any assessment of risk of bias that may affect the cumulative evidence (e.g., publication bias, selective reporting within studies). | *Each study was critically appraised separately by using design-specific critical appraisal tools from the UK National Institute for Health and Care Excellence (NICE) [13]. The study´s quality was described as follows: “(++) All or most of the checklist criteria have been fulfilled, where they have not been fulfilled the conclusions are very unlikely to alter. (+) Some of the checklist criteria have been fulfilled, where they have not been fulfilled, or not adequately described, the conclusions are unlikely to alter. (-) Few or no checklist criteria have been fulfilled and the conclusions are likely or very likely to alter.” [13]. Mixed methods were analysed by the Mixed Methods Appraisal Tool (MMAT) – Version 2011 [14]. The critical appraisal for mixed-methods studies includes whether the mixed-methods design was appropriate, and whether the integration was relevant to address the research question (objective). The criteria also consider whether limitations are considered, associated with this integration, e.g. whether the divergence of qualitative and quantitative data (or results) in a triangulation design was appropriate [14].* | 11 |
| Additional analyses | 16 | Describe methods of additional analyses (e.g., sensitivity or subgroup analyses, meta-regression), if done, indicating which were pre-specified. | *Not applicable* | / |
| **RESULTS** | | |  |  |
| Study selection | 17 | Give numbers of studies screened, assessed for eligibility, and included in the review, with reasons for exclusions at each stage, ideally with a flow diagram. | *In total, 1,993 publications were identified that had been published up to June 2015, of which 26 publications (n=25 studies) reporting diabetes-related IN of people with DM were finally included.*  *A flow diagram is provided in figure 1.* | 12, figure 1 |
| Study characteristics | 18 | For each study, present characteristics for which data were extracted (e.g., study size, PICOS, follow-up period) and provide the citations. | *The study characteristics are provided in table 1.* | Table 1 |
| Risk of bias within studies | 19 | Present data on risk of bias of each study and, if available, any outcome level assessment (see item 12). | *The results of the critical appraisals are shown in table 1.* | table 1 |
| Results of individual studies | 20 | For all outcomes considered (benefits or harms), present, for each study: (a) simple summary data for each intervention group (b) effect estimates and confidence intervals, ideally with a forest plot. | *The study characteristics are provided in table 1.* | Table 1 |
| Synthesis of results | 21 | Present results of each meta-analysis done, including confidence intervals and measures of consistency. | *The study characteristics are provided in table1.* | Table 1 |
| Risk of bias across studies | 22 | Present results of any assessment of risk of bias across studies (see Item 15). | *The critical appraisal showed that three of the 25 identified studies met all or most of the NICE checklist criteria. The other studies fulfilled some (n=14) or a few criteria (n=8). It was noticeable that within the qualitative studies most of the criteria were fulfilled but eight of 14 studies did not describe the role of the researcher sufficiently, and six gave no indications concerning ethical approval. None of the included quantitative studies reported how selection bias was minimised, and included studies using mixed-method design reported little about the quantitative part of their study design. The results of the critical appraisals are shown in table 1.* | 13, table 1 |
| Additional analysis | 23 | Give results of additional analyses, if done (e.g., sensitivity or subgroup analyses, meta-regression [see Item 16]). | *Thirteen studies investigated IN as a primary outcome (primary research aim). Twelve of these analysed the type of information needed by people with DM, and one study investigated the relationships between IN, diagnosis and disease (table 1). The other studies (n=12) reported IN as a secondary outcome and focused predominantly on other topics, e.g. information exchange, patient experience and information sources. Altogether, we identified 14 qualitative studies, six quantitative studies and five mixed-method studies. Four different methods were reported throughout: interviews, group methods, surveys using written questionnaires and website evaluation.* | 12, table 1 |
| **DISCUSSION** | | |  |  |
| Summary of evidence | 24 | Summarize the main findings including the strength of evidence for each main outcome; consider their relevance to key groups (e.g., healthcare providers, users, and policy makers). | *This is the first systematic review of studies dealing with IN of people with DM. We identified 25 studies. This is a limited number compared with, say, cancer, where a large number of quantitative and qualitative IN studies (n=112) already exist [39]. This is surprising, since it is known that people with DM have a higher or similarly high need for information compared with people with other chronic diseases [3, 4]. Looking for the content of IN, it was comparable to those found in people with cancer, such as ʻprognosis of diseaseʼ, ʻdiagnostic testsʼ, ʻtreatmentʼ, ʻself-careʼ, ʻemotional and psychological needsʼ [9]. However, Duggan et al. (2008) found that people with DM have a higher need for information about drugs than people with cancer or cardiovascular disease [4]. Besides the low number of studies, it became obvious that differences between patient groups such as male and female patients, different age groups or types of diabetes have not been analysed so far. Factors associated with IN are rarely investigated. In cases in which analysis was performed, mainly the more complex factors such as participation preferences or information seeking were investigated, and it was particularly done in qualitative studies, which were highly heterogeneous. Also, changes in IN during the course of the disease are poorly investigated, although they may be expected.*  *Instruments for collecting IN data from individuals with DM have not been validated. Similar results were identified in cancer studies and showed that only a minority of instruments for the collection of IN are validated* | 20, 21 |
| Limitations | 25 | Discuss limitations at study and outcome level (e.g., risk of bias), and at review-level (e.g., incomplete retrieval of identified research, reporting bias). | *We conducted a comprehensive and sensitive search that was also pre-tested. The study selection and critical appraisal were performed by two reviewers. Two other reviewers checked all the decisions. The critical appraisals were performed for each study design. However, there were still difficulties to provide a clear decision based on the reports.*  *The identified studies have several limitations: only two qualitative studies met all the quality criteria. None of the quantitative studies met all the criteria. The comparability of the IN categories is restricted because the IN were collected by different study designs. Some of them were collected in the context of a specific health-related topic or by using different methods or instruments.* | 21 |
| Conclusions | 26 | Provide a general interpretation of the results in the context of other evidence, and implications for future research. | *There is a limited number of studies analysing IN in DM, and there is a low number of studies investigating differences between subgroups of DM populations, including gender aspects or changes of information needs during the disease. This should be further investigated.* | 22 |
| **FUNDING** | | |  |  |
| Funding | 27 | Describe sources of funding for the systematic review and other support (e.g., supply of data); role of funders for the systematic review. | *This work was supported by the Research Committee of the Heinrich Heine University [9772577]* |  |

*From:*  Moher D, Liberati A, Tetzlaff J, Altman DG, The PRISMA Group (2009). Preferred Reporting Items for Systematic Reviews and Meta-Analyses: The PRISMA Statement. PLoS Med 6(7): e1000097. doi:10.1371/journal.pmed1000097

For more information, visit: **www.prisma-statement.org**.

Page 2 of 2

**References:**

1. Mühlhauser I. Diabetes experts' reasoning about diabetes prevention studies: a questionnaire survey. BMC Research Notes. 2008;1:90.

2. Ahola AJ, Groop PH. Barriers to self-management of diabetes. Diabet Med. 2013;30:413-20.

3. Beeney LJ, Bakry AA, Dunn SM. Patient psychological and information needs when the diagnosis is diabetes. Patient Educ Couns.1996,29:109-16.

4. Duggan C, Bates I. Medicine information needs of patients: the relationships between information needs, diagnosis and disease. Qual Saf Health Care 2008;17:85-9.

5. Moher D, Liberati A, Tetzlaff J, Altman DG, PRISMA Group. Preferred reporting items for systematic reviews and meta-analyses: the PRISMA statement. PLoS Med. 2009;7:e1000097.

6. National Institute for Health and Care Excellence. Assessing the quality of evidence. In Methods for the Development of NICE Public Health Guidance (third edition). National Institute for Health and Care Excellence (NICE). 2012;72-3.

7. Pluye P, Gagnon MP, Griffiths F, Johnson-Lafleur J. A scoring system for appraising mixed methods research, and concomitantly appraising qualitative, quantitative and mixed methods primary studies in Mixed Studies Reviews. Int J Nurs Stud. 2009; 46:529-46.

8. Finfgeld-Connett D. Use of content analysis to conduct knowledge-building and theory-generating qualitative systematic reviews. Qualitative Research 2014; doi: 10.1177/1468794113481790.

9. Rutten LJ, Arora NK, Bakos AD, Aziz N, Rowland J. Information needs and sources of information among cancer patients: a systematic review of research (1980-2003). Patient Educ Couns. 2005;57:250-61.
